# Mass Transfer Principles in Column Percolation Tests: Initial Conditions and Tailing in Heterogeneous Materials

**DOI:** 10.3390/ma14164708

**Published:** 2021-08-20

**Authors:** Binlong Liu, Michael Finkel, Peter Grathwohl

**Affiliations:** Center for Applied Geoscience, University of Tübingen, Schnarrenbergstraße 94-96, 72076 Tübingen, Germany; binlong.liu@uni-tuebingen.de (B.L.); michael.finkel@uni-tuebingen.de (M.F.)

**Keywords:** leaching test, equilibrium condition, non-equilibrium condition, modelling, sorption kinetics, non-linear sorption, heterogeneity

## Abstract

Initial conditions (pre-equilibrium or after the first flooding of the column), mass transfer mechanisms and sample composition (heterogeneity) have a strong impact on leaching of less and strongly sorbing compounds in column percolation tests. Mechanistic models as used in this study provide the necessary insight to understand the complexity of column leaching tests especially when heterogeneous samples are concerned. By means of numerical experiments, we illustrate the initial concentration distribution inside the column after the first flooding and how this impacts leaching concentrations. Steep concentration gradients close to the outlet of the column have to be expected for small distribution coefficients ($K_{d}<1$ L kg^−1^) and longitudinal dispersion leads to smaller initial concentrations than expected under equilibrium conditions. In order to elucidate the impact of different mass transfer mechanisms, film diffusion across an external aqueous boundary layer (first order kinetics, FD) and intraparticle pore diffusion (IPD) are considered. The results show that IPD results in slow desorption kinetics due to retarded transport within the tortuous intragranular pores. Non-linear sorption has not much of an effect if compared to $K_{d}$ values calculated for the appropriate concentration range (e.g., the initial equilibrium concentration). Sample heterogeneity in terms of grain size and different fractions of sorptive particles in the sample have a strong impact on leaching curves. A small fraction (<1%) of strongly sorbing particles (high $K_{d}$) carrying the contaminant may lead to very slow desorption rates (because of less surface area)—especially if mass release is limited by IPD—and thus non-equilibrium. In contrast, mixtures of less sorbing fine material (“labile” contamination with low $K_{d}$), with a small fraction of coarse particles carrying the contaminant leads to leaching close to or at equilibrium showing a step-wise concentration decline in the column effluent.

## 1. Introduction

Leaching tests are widely used for the determination of contaminant release rates from soils [1,2,3,4], recycling materials [5,6,7,8,9,10,11], construction products [12,13,14,15,16], radioactive and other waste materials [17,18]. Compared to traditional batch shaking tests, column tests are preferred for assessing the risk of release of potential pollutants into groundwater or surface waters because they are closer to natural conditions [19,20]. Different mechanisms controlling desorption kinetics may result in complex leaching behaviors. While initially the observed column effluent concentration often reflects equilibrium conditions between the solid phase (incl. intraparticle pores) and the mobile aqueous phase [21,22], the concentrations decrease and often an extended tailing is observed due to slow desorption processes such as intraparticle diffusion [23,24,25].

Although most leaching test procedures aim at equilibrium conditions in the column before the leaching starts, the true concentration distribution before the start of the percolation depends not only on the test procedure (contact time, pre-equilibration time, flow velocity during first flooding) but also on the properties of both the solid material and the pollutant of interest [26]. Equilibration and long-term leaching are further complicated if the test sample consists of a heterogeneous mixture of different material types and grain sizes [26,27,28], which is the common case if waste materials such as demolition waste or soils are tested.

Finkel and Grathwohl (2017) evaluated the role of initial conditions for column leaching tests with intraparticle pore diffusion models by comparing the hypothetical scenarios, a perfectly equilibrated column vs. a column that wasn’t equilibrated at all [26]. They could show that in many practical cases, peak and cumulative leachate concentrations are rather independent of the initial conditions. However, if release kinetics are slow due to large grain size or small intragranular porosity, the sensitivity to initial conditions is relevant, in particular for initial peak and early cumulative leachate concentrations. 

The shortcomings of all previous studies [26,29,30], is that only uniform initial concentrations in the columns were considered in the leaching models. However, due to the specific conditions during the flooding of the column filled with initially dry material, the true initial conditions at the start of the leaching test may considerably deviate from this ideal, i.e., uniform distribution.

Against this background, the objectives of this study are (i) to illustrate the possibly non-uniform initial conditions that may be achieved after the first flooding of the column, (ii) to show the impact of these initial conditions on the temporal development of the effluent concentrations, and (iii) to investigate how heterogeneous mixtures of particles having different properties affect both the initial conditions in the column and the leaching of the solutes. To achieve that, we used numerical solutions for flow and transport in a column coupled to two kinetic models: (i) solute diffusion through an aqueous boundary layer and (ii) intraparticle pore diffusion. The implementation of the numerical models is described in detail in the appendices.

## 2. Theory and Background

### 2.1. Local Equilibrium: The Advection—Dispersion Equation

In order to facilitate the understanding of mass transfer-limited cases of contaminant release in a column, we briefly introduce the equilibrium case for which the advection-dispersion model is commonly used:

(1) $$\frac{\partial }{\partial t}\left(n\;C_{w}+\rho_{b}\;C_{s}\right)+\frac{\partial }{\partial x}\left(n\;v\;C_{w}-n\;D_{L}\frac{\partial C_{w}}{\partial x}\right)=0$$

where v [L T^−1^], n [-] and $D_{L}\;$($=\alpha v+D_{p})$ [L^2^ T^−1^] denote the seepage velocity of the water, the intergranular porosity and the longitudinal dispersion coefficient. α [L], $D_{p}$ (=$nD_{aq}$) [L^2^ T^−1^] and $D_{aq}$ [L^2^ T^−1^] denote the dispersivity, the pore diffusion coefficient and the aqueous diffusion coefficient of the solute. x [L] and t [T] are the length of the column and time. $\rho_{b}\;$($=\left(1-n\right)\rho_{s}$) [M L^−3^] is the dry bulk density of the packed bed in the column (porous media; $\rho_{s}$ is the solids density). For local equilibrium conditions the concentration in the solid phase $\left(C_{s}\right)$ is in equilibrium with the solute concentration in water $\left(C_{w}\right)$ and the distribution coefficient $K_{d}\;(=C_{s}/C_{w})$ allowing for the calculation of the respective concentrations. Under these conditions, Equation (1) can be simplified as:

(2) $$\frac{\partial C_{w}}{\partial t}=\frac{D_{L}}{R_{d}}\frac{\partial^{2}C_{w}}{\partial x^{2}}-\frac{v}{R_{d}}\frac{\partial C_{w}}{\partial x}$$

where $R_{d}$ [-] represents the retardation factor, defined as:

(3) $$R_{d}=1+K_{d}\frac{\rho_{b}}{n}$$

Assuming equilibrium conditions and an initially uniform distribution of the solute in the column, leaching may be described by the analytical solution of Equation (2) [31]:

(4) $$\frac{C_{w}}{C_{w,eq}}=1-0.5\left[\mathrm{erfc}\left(\frac{x-\frac{v}{R_{d}}t}{2\sqrt{\frac{D_{L}}{R_{d}}t}}\right)+\exp\left(\frac{v\;x}{D_{L}}\right)\mathrm{erfc}\left(\frac{x+\frac{v}{R_{d}}t}{2\sqrt{\frac{D_{L}}{R_{d}}t}}\right)\right]$$

where erfc denotes the complementary error function. The aqueous concentration at equilibrium, $C_{w,eq}$, can be calculated from the initial solid concentration ($C_{s,ini}$) accounting for the mass balance when the contaminant mass in the water is equilibrated with the mass in the solid:

(5) $$C_{w,eq}=\frac{C_{s,ini}}{K_{d}+\frac{n}{\rho_{b}}}$$

**Figure 1:**
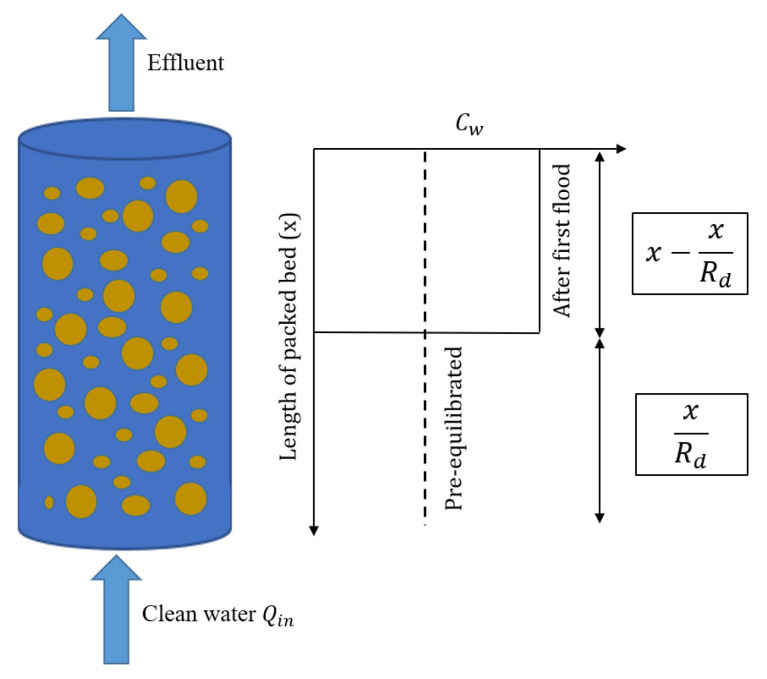
Initial concentration distribution in a column of length x for the “pre-equilibrated” case (dashed line) and after the first flooding of an initially dry column from the bottom (solid line); no dispersion, $R_{d}=2$, after Grathwohl and Susset, 2009 [21].

The ratio $n/\rho_{b}$ [L^3^ M^−1^] equals the liquid to solid ratio within the column, which in most cases is much smaller than in a batch leaching test (e.g., 0.25 L kg^−1^ for a column test with a porosity of n= 0.4 and a solid density of $\rho_{s}=$ 2.65 g cm^−3^, compared to e.g., 10 L kg^−1^ in a batch test). Since leaching tests start for practical reasons with material packed more or less dry into the column, a uniform initial concentration is not necessarily achieved during the first flooding of the column. Initial conditions as assumed in Equation (4) (uniform concentration distribution), would only be achieved if the material is first mixed with water, equilibrated and then packed into the column, which is not practical. During the first flooding of the column, especially less sorbing solutes are displaced from the inlet and higher concentrations occur towards the outlet, as illustrated in Figure 1 (see also Appendix E). This may be accounted for by subtracting the distance of the solute displaced initially ($x/R_{d}$ with $R_{d}$ > 1) in Equation (4):

(6) $$\begin{array}{cc} \frac{C_{w}}{C_{w,eq}}=1-0.5 & [\mathrm{erfc}(\frac{\left(x-\frac{x}{R_{d}}\right)-\frac{v}{R_{d}}t}{2\sqrt{\frac{D_{L}}{R_{d}}t}})\\ & +\exp\left(\frac{v\left(x-\frac{x}{R_{d}}\right)}{D_{L}}\right)\mathrm{erfc}\left(\frac{\left(x-\frac{x}{R_{d}}\right)+\frac{v}{R_{d}}t}{2\sqrt{\frac{D_{L}}{R_{d}}t}}\right)] \end{array}$$

In this case the initial solute concentration ($C_{w,eq}$) in the column effluent is in equilibrium with the initial concentration in the solids ($C_{w,eq}=C_{s,ini}/K_{d}$) and higher than calculated in Equation (5), especially if $K_{d}$ values are low.

In order to explore the influence of mass transfer limitations on initial and long-term solute concentrations in column tests, two relevant mass transfer mechanisms are compared: (1) film diffusion where diffusion from the solid-water interface occurs through an aqueous boundary layer with a given thickness and (2) intraparticle pore diffusion where diffusion inside the porous particle limits mass transfer.

### 2.2. Desorption Kinetics Limited by Film Diffusion

**Figure 2:**
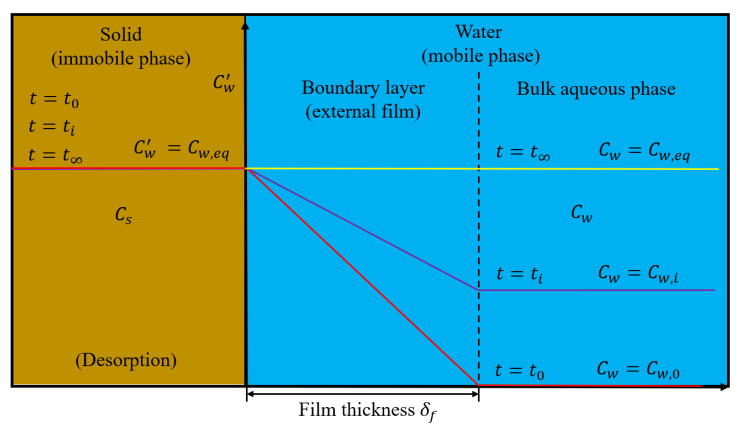
Scheme of mass transfer limited by film diffusion during the first flooding with fixed concentration at the interface (because the infiltrating water is always contacting fresh material as it advances).

The simplest model for the kinetic release of a solute from solids considers diffusion through an aqueous boundary layer surrounding spherical particles (Figure 2). Such film diffusion models are also widely used for the dissolution of minerals with high solubilities (e.g., salts). The solute release from the solid surface into the bulk water phase can be described by a linear driving force with constant mass transfer coefficient $k=D_{aq}/\delta_{f}$:

(7) $$\frac{\partial C_{w}}{\partial t}=k\;A^{o}\left(C_{w}^{\prime }-C_{w}\right)=\frac{D_{aq}}{\delta_{f}}\frac{m_{d}\;6}{\rho_{s}\;d\;V_{w}\;}\left(C_{w}^{\prime }-C_{w}\right)$$

where $\delta_{f}\;\left[L\right]$, $V_{w}$ [L^3^], $m_{d}$ [M] and d [L] denotes the thickness of the external film, the volume of water, the dry mass of the solids in the column and the particle diameter, respectively. $A^{o}\;$($=6\;m_{d}$/($V_{w}\;\rho_{s}\;d$)) is the specific surface area of the particles per unit volume of water in the column [m^2^ m^−3^ = m^−1^] (the term $6/\rho_{s}\;d$) represents the specific surface area of spherical particles per dry mass, e.g., in m^2^ g^−1^). $C_{w}^{\prime }$ is the concentration at the solid-water interface where local equilibrium conditions apply ($C_{w}^{\prime }=C_{s}/K_{d}$). The external film thickness ($\delta_{f}$) can be estimated from empirical Sherwood numbers (Sh) and the particle diameter (d):

(8) $$Sh=\frac{kd}{D_{aq}}=\frac{d}{\delta_{f}}\;\to \;\delta_{f}=\frac{d}{Sh}$$

For an overview on empirical relationships for the estimation of Sherwood numbers see Appendix A. The mass balance in such two-phase systems expressed by their respective rates is:

(9) $$V_{w}\frac{\partial C_{w}}{\partial t}=-m_{d}\frac{\partial C_{s}}{\partial t}$$

Thus, the solute mass gained (or lost) by the water phase equals the solute mass lost (or gained) from the solid phase. If $V_{w}$ and $m_{d}$ in a packed bed (porous media) are replaced by n and $\rho_{b}$, the density of the solids ($\rho_{s}$) in Equation (7) drops out. Film diffusion coupled to the one-dimensional advection-dispersion equation (Equation (1)) yields:

(10) $$\frac{\partial C_{w}}{\partial t}=D_{L}\frac{\partial^{2}C_{w}}{\partial x^{2}}-v\frac{\partial C_{w}}{\partial x}+\frac{D_{aq}}{\delta_{f}}\frac{6\;\left(1-n\right)}{n\;d}\left(\frac{C_{s}}{K_{d}}-C_{w}\right)$$

**Figure A1:**
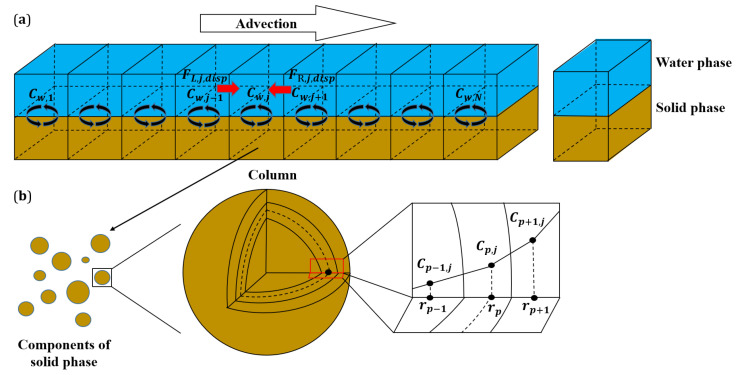
Discretization of the column into *N* parts (**a**). Representation of the solid phase as a composition of grains having different sizes and properties, each discretized by a number of *L* shells (**b**).

Using the finite volume method, the column is spatially discretized by a number of cells (see Figure A1) and the governing equation (Equation (10)) is solved iteratively by employing the Newton–Raphson scheme. Details of the numerical solution of the film diffusion model are presented in Appendix B.

### 2.3. Desorption Limited by Intraparticle Pore Diffusion 

**Figure 3:**
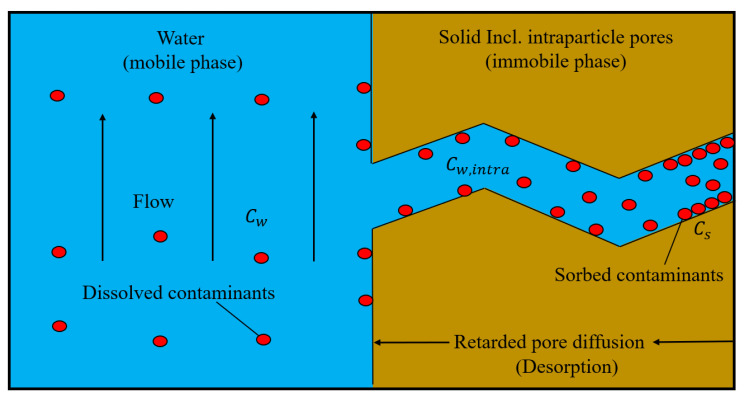
Scheme of mass transfer limited by intraparticle pore diffusion.

If the release of compounds from the solid phase is governed by intra-granular diffusion, e.g., within a porous grain (Figure 3), then mass transfer is described by Fick’s second law in radial coordinates:

(11) $$\frac{\partial }{\partial t}\left(\varepsilon \;C_{w,intra}+\rho_{p}C_{s}\right)=D_{e}\left[\frac{\partial^{2}C_{w,intra}}{\partial r^{2}}+\frac{2}{r}\frac{\partial C_{w,intra}}{\partial r}\right]$$

with the boundary conditions

(12) $$C_{w,intra}\left(r=R,t\right)=C_{w}$$

(13) $$\frac{\partial C_{w,intra}}{\partial r}\left(r=0,t\right)=0$$

*r* [L] is the radial coordinate in the sphere and $D_{e}$ [L^2^ T^−1^] the effective diffusion coefficient. $C_{w,intra}$ [M L^−3^] is the concentration of solute in the intra-granular pore water. ε [-] denotes the intraparticle porosity. R [L] and $\rho_{p}$ [M L^−3^] ($=\rho_{s}\left(1-\varepsilon \right))$ denote the radius and bulk density of the particle (sphere). 

For linear sorption with concentration independent distribution coefficients (i.e., $C_{s}=K_{d}\;C_{w,intra}$) Equation (11) becomes:

(14) $$\frac{\partial C_{w,intra}}{\partial t}=D_{a}\left[\frac{\partial^{2}C_{w,intra}}{\partial r^{2}}+\frac{2}{r}\frac{\partial C_{w,intra}}{\partial r}\right]$$

where $D_{a}$ [L^2^ T^−1^] is the apparent diffusion coefficient, defined as:

(15) $$D_{a}=\frac{D_{e}}{\varepsilon +K_{d}\rho_{p}}=\frac{D_{aq}\varepsilon }{\tau \left(\varepsilon +K_{d}\rho_{p}\right)}\approx \frac{D_{aq}\varepsilon^{2}}{\varepsilon +K_{d}\left(1-\varepsilon \right)\rho_{s}}$$

Empirical studies showed that $D_{e}$ increases approximately with the square of the porosity [32]. This corresponds to a tortuosity τ [-] of the intra-granular pores—if expressed as a function of intra-granular porosity—of τ≈1/ε.

Considering intraparticle diffusion, the advection-dispersion model (Equation (1)) can be rewritten as:

(16) $$\frac{\partial }{\partial t}\left(nC_{w}+\left(1-n\right)\left(\varepsilon C_{w,intra}+\rho_{p}C_{s}\right)\right)+\frac{\partial }{\partial x}\left(nvC_{w}-nD_{L}\frac{\partial C_{w}}{\partial x}\right)=0$$

The corresponding equilibrium concentration ($C_{w,eq}$) in water after first flooding can be rewritten as $C_{w,eq}=C_{s,ini}/(\varepsilon /\rho_{p}+K_{d})$, which is slightly lower than for non-porous solids ($C_{w,eq}=C_{s,ini}/K_{d}$) because the intragranular pore space is assumed to be initially free of water. The deviation becomes insignificant with the increase of $K_{d}$ ($\varepsilon /\rho_{p}+K_{d}\approx K_{d}$ when $K_{d}\gg \varepsilon /\rho_{p}$). 

Coupling the intraparticle pore diffusion model (Equation (11)) to the one-dimensional advection-dispersion equation (Equation (16)) allows for the expression of the change of the solute concentration in the bulk water:

(17) $$\frac{\partial C_{w}}{\partial t}=D_{L}\frac{\partial^{2}C_{w}}{\partial x^{2}}-v\frac{\partial C_{w}}{\partial x}-\frac{\left(1-n\right)}{n}D_{e}\left[\frac{\partial^{2}C_{w,intra}}{\partial r^{2}}+\frac{2}{r}\frac{\partial C_{w,intra}}{\partial r}\right]$$

The intraparticle pore diffusion model (Equations (11)–(13)) was implemented numerically using a finite volume method where the spherical particle was discretized by a number of spherical shells of equal volume (based on the method of Jäger and Liedl [28]). The column was spatially discretized by a number of cells (see Figure A1) and all the governing equations (Equations (11)–(13) and (17)) were solved iteratively applying the Newton–Raphson scheme. Compared to the 1D film diffusion case, the intraparticle pore diffusion case is more complicated and becomes a 2D problem. Details of the numerical solution of the intraparticle pore diffusion model are given in Appendix C.

### 2.4. Set-Up of “Numerical” Column Tests

The boundary conditions of the numerical experiments are based on the set-up of column tests in daily practice in Germany [21,33,34]. Table 1 lists the relevant material properties and the parameter ranges applied. The saturation time for the first pore volume of the column and the contact time (after the first flooding period) were set to 5 h. Initially, experiments with uniform materials were simulated where the intraparticle porosity, distribution coefficient, aqueous diffusion coefficient and tortuosity were set the same for each grain size fraction. The Sherwood number in packed beds was estimated based on the empirical formula proposed by Liu et al. (2014) (Equation (A3)) [35]. In order to illustrate the influence of longitudinal dispersion on the initial concentration distribution in the column after the first flooding, we used fine particles ($d_{p,\;fine}=63\;\mu m$) where kinetics are very fast, and the local equilibrium assumption is valid. The numerical model did not consider dispersion beyond the outlet of the column. Non-linear sorption was simulated using the Freundlich model ($C_{s}=K_{fr}C_{w}^{\frac{1}{n}}\;$ where $K_{fr}$ and 1/*n* denote the Freundlich coefficient and Freundlich exponent, respectively). 

Many factors may contribute to sample heterogeneity, such as grain size distribution and particle properties (sorption, porosities, etc.). To highlight the impact of particle size and properties we focused on two grain size classes and different fractions of sorptive/reactive particles in the sample. Distribution coefficients were varied in a range of 0.1–100 L kg^−1^. Lower $K_{d}$ values (<0.1 L kg^−1^) were not considered here (this would have resulted in very high initial aqueous concentrations). If the $K_{d}$ values become large ($K_{d}$ > 100 L kg^−1^), then the differences between the pre-equilibrated case and the “first flooding” scenario vanish and effluent concentrations are constant over long time periods. The $K_{d}$ range chosen covers many frequent environmental contaminants, such as per- and polyfluoroalkyl substances (PFAS), chlorinated solvents, polycyclic aromatic hydrocarbons and some heavy metals.

## 3. Results and Discussion

### 3.1. Impact of Initial Conditions on Leaching 

**Figure 4:**
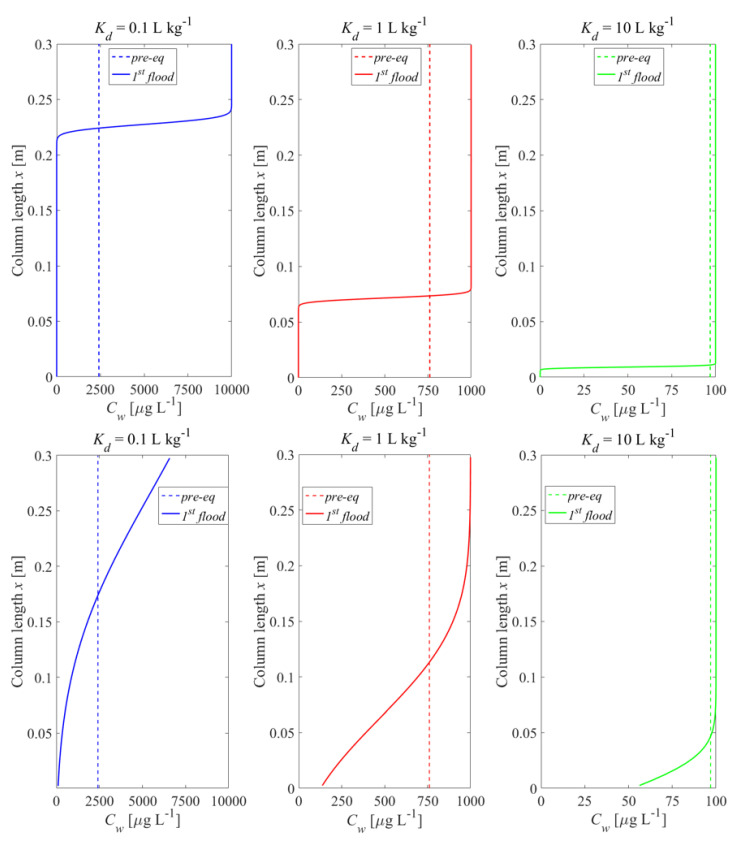
Initial aqueous concentration distributions in the column after the first flooding (solid lines) if mass transfer is controlled by film diffusion for three different distribution coefficients, $K_{d}$, compared to the pre-equilibrated case (dashed lines). **Top panel**: without dispersion; **bottom panel**: with dispersion; *n* = 0.45, *v* = 1.67 × 10^−5^ m s^−1^, α/*x* = 0 or 0.1, $C_{s,ini}$ = 1000 μg kg^−1^, $t_{c}$ = 5 h, $d_{p,fine}$ = 63 μm.

In order to investigate the impact of initial conditions on leaching behavior, we compared two scenarios: (1) a column filled with pre-equilibrated material where the initial concentration distribution in the column was uniform ($C_{w,eq}=C_{s,ini}/\left(K_{d}+\frac{n}{\rho_{b}}\right)$) and (2) columns with non-uniform concentration distributions after first flooding where concentrations increased towards the outlet (to a maximum of $C_{w,eq}=C_{s,ini}/K_{d}$) while at the inlet the solute was already depleted. To illustrate this, we used the film diffusion model with fine grain sizes, and thus, fast kinetics (local equilibrium conditions). Figure 4 shows the initial aqueous concentration distribution in the up-flow column test after the first flooding of the column compared to the pre-equilibrium case. The results show that the differences in the initial concentration profiles became smaller with increasing sorption. At high $K_{d}$ values, the deviation of the initial concentration profiles only occurred at the inlet of the column. At low $K_{d}$ values, very high concentrations are expected at the column outlet; in extreme cases this may lead to a density increase in the leachate and—especially if flow is stopped—to density driven flow within the column. This would cause dilution and lower leachate concentrations when the flow is restarted as was potentially observed by Naka et al. [36]. Density driven mixing may be caused by soluble materials, e.g., sulphate or other salts and not necessarily the target compounds. This phenomenon is quite similar to the case where the dispersion is taken into account (see bottom curves of Figure 4 and also Figures S1–S8 in SM), which leads to more “mixing” in the column and thus lower initial concentrations at the outlet, especially for low $K_{d}$ values. 

**Figure 5:**
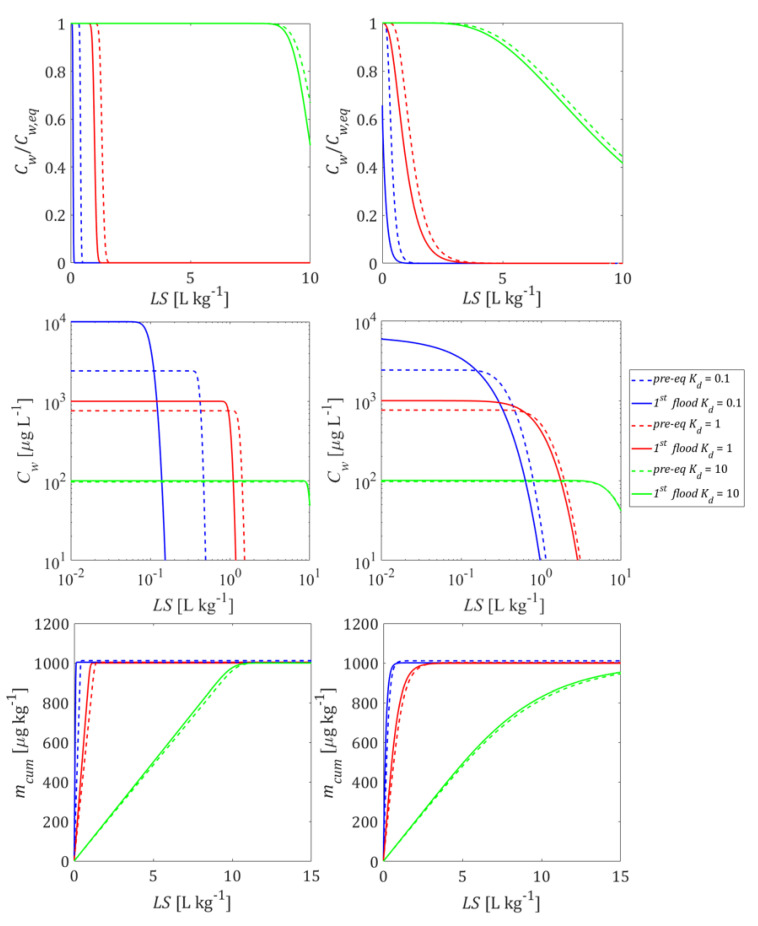
Normalized and absolute concentration ($C_{w}/C_{w,eq},C_{w}$) as well as cumulative concentration ($m_{cum}$ ) in the column effluent vs. time (expressed as liquid to solid ratio: *LS*) for the initial conditions (depicted in Figure 4) established after the first flooding of the column (solid lines) compared to the pre-equilibrated case (dashed lines). **Left column**: without dispersion; **right column**: with dispersion.

Figure 5 shows how the initial conditions (pre-equilibrated column and column after first flooding) influence the leaching curves. Compared to the pre-equilibrated case, a higher equilibrium concentration appeared at the outlet of the column after the first flooding period and more contaminant mass was released from the column at early times, followed by a rapidly decreasing concentration (see Figure 5, 2nd row). The deviations vanished with increasing $K_{d}$ and became almost insignificant for $K_{d}\geq \;$10 L kg^−1^. Dispersion also reduces differences between the pre-equilibrated and the first flooding case. At high $K_{d}$ values, the maximum concentrations were still achieved but the tailings became smoother. With the decrease of the $K_{d}$ value, the concentration gradients at the inlet became steeper and the “back” dispersion fluxes towards the outlet increased as well. In extreme cases, the peak concentration at the column outlet was smaller than the maximum concentration expected (e.g., $\;K_{d}=$ 0.1 L kg^−1^). The effect of initial conditions on normalized concentrations looks like a phase shift (see Figure 5, 1st row). This would lead to an underestimation of $K_{d}$ values derived from the pre-equilibrium analytical solution (Equation (4)) if the conditions in the column after the first flooding are not appropriately considered. The lower the $K_{d}$, the earlier the cumulative leachate concentration reaches its maximum value ($m_{cum,\;max}=1000\;\mu$g kg^−1^). Dispersion shifts this point to later times (see Figure 5, 3rd row).

### 3.2. Initial Conditions and Leaching with Mass Transfer Limitations

**Figure 6:**
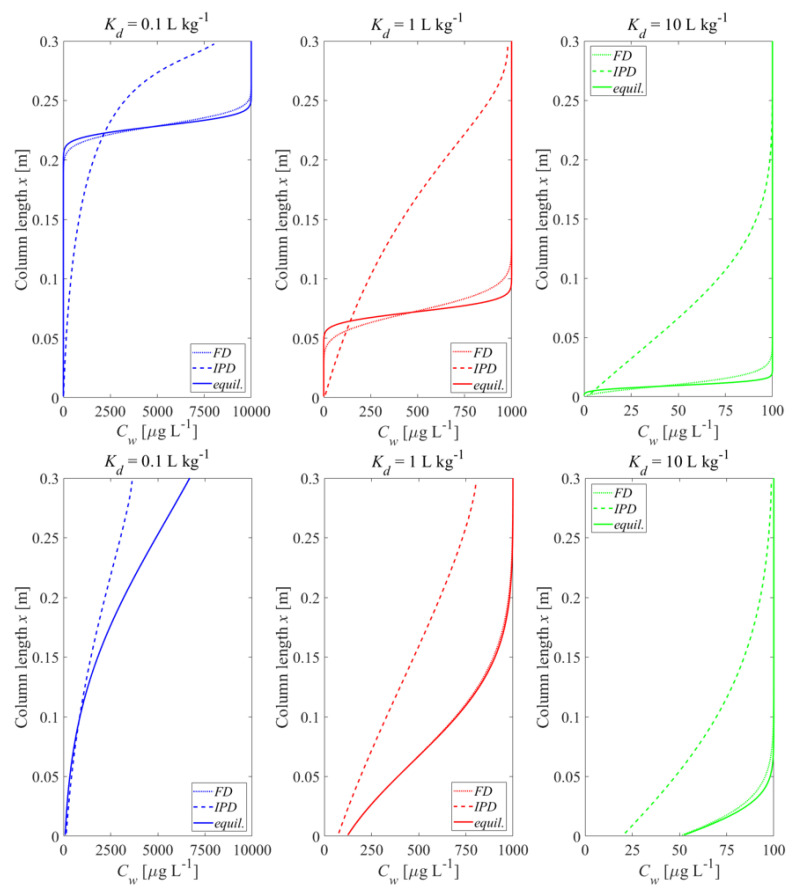
Initial aqueous concentration distributions in the column after the first flooding depending on the mass transfer limitation; dotted lines: film diffusion (FD), dashed lines: intraparticle diffusion (IPD); solid lines: fast kinetics (equilibrium, fine particles). **Top panel**: without dispersion; **bottom panel**: with dispersion; *n* = 0.45, *v* = 1.67 × 10^−^^5^ m s^−^^1^, α/*x* = 0 or 0.1, $C_{s,ini}$ = 1000 μg kg^−^^1^, $t_{c}$ = 5 h, $D_{aq}$ = 1 × 10^−^^9^ m^2^ s^−^^1^, ε = 0.05, $d_{p,coarse}$ = 2000 μm, $d_{p,fine}$ = 63 μm.

**Figure A2:**
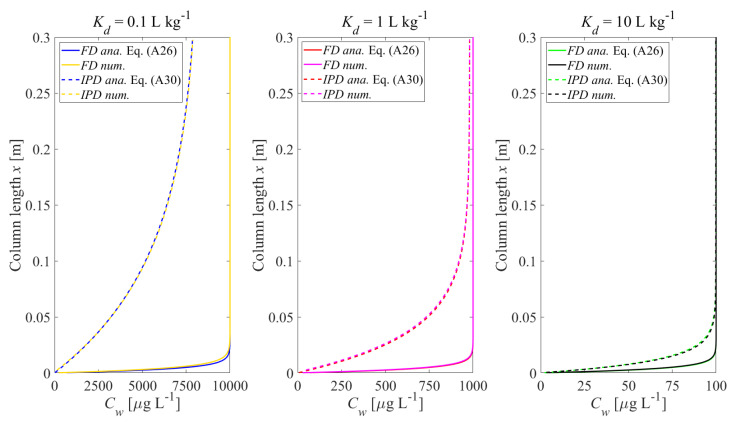
Concentration increase in a water parcel ($C_{w,peak}$) in the column during the first flooding (up-flow); solid lines: film diffusion; dashed lines: intraparticle diffusion; comparison between analytical (ana.) and numerical (num.) solutions. *n* = 0.45, *v* = 1.67 × 10^−^^5^ m s^−^^1^, α /*x* = 0 (no dispersion), $C_{s,ini}$ = 1000 μg kg^−^^1^, t = 5 h, $D_{aq}$ = 1 × 10^−^^9^ m^2^ s^−^^1^, ε = 0.05, $d_{p,coarse}$ = 2000 μm.

Mass-transfer limitations may change the picture considerably, with respect to initial conditions and the development of leachate concentrations over time. Figure 6 shows the influence of film diffusion (FD) compared to intraparticle pore diffusion (IPD) limited desorption on the initial concentration distribution in the column after the first flooding period. For large $K_{d}$ values, equilibration is achieved after shorter distances in the column because of the retardation of the clean water front. The deviations between FD and IPD are due to different mass transfer zone lengths, $X_{s,63.2\%}$ (see Appendix D for a discussion of the concept and calculation of this length for FD and IPD). For FD, the mass transfer zone length is independent of the $K_{d}$ value and proportional to the particle size (Equation (A28)). In contrast, for IPD the length of the mass transfer zone increases with particle size to the power of 3/2 ($d^{3/2}$) and decreases with $\sqrt{K_{d}}$ (Equation (A32)) (e.g., $X_{s,63.2\%}=$ 10 cm, 3.5 cm, and 1.1 cm for $K_{d}$ values of 0.1 L kg^−1^, 1 L kg^−1^ and 10 L kg^−1^ (see Figure A2)). The length of the mass transfer zone for IPD is much longer than for FD, but differences decrease with increasing $K_{d}$ values. For $K_{d}$ values of 1 L kg^−1^ and 10 L kg^−1^, the mass transfer zone lengths for IPD are much shorter than the column length ($X_{col}$ = 30 cm), which indicates that the equilibrium concentration is achieved at the outlet of the column after the first flooding. For small $K_{d}$ values (e.g., $K_{d}=$ 0.1 L kg^−1^), the equilibrium concentrations are not achieved at the outlet if dispersion is considered (see Figure 6, lower panel) although the mass transfer zone length ($X_{s,63.2\%}=10\;\mathrm{cm}$) is still shorter than the column length. This is because the “clean” water front is close to the column outlet and dispersion “dilutes” the steep concentration gradients (“back dispersion”). The deviations between FD and fast kinetics almost vanish when dispersion is considered, indicating that with film diffusion, equilibrium is almost achieved. The development of the concentration distribution for IPD is also illustrated in animated graphs provided in the Supplementary Material (SM).

**Figure 7:**
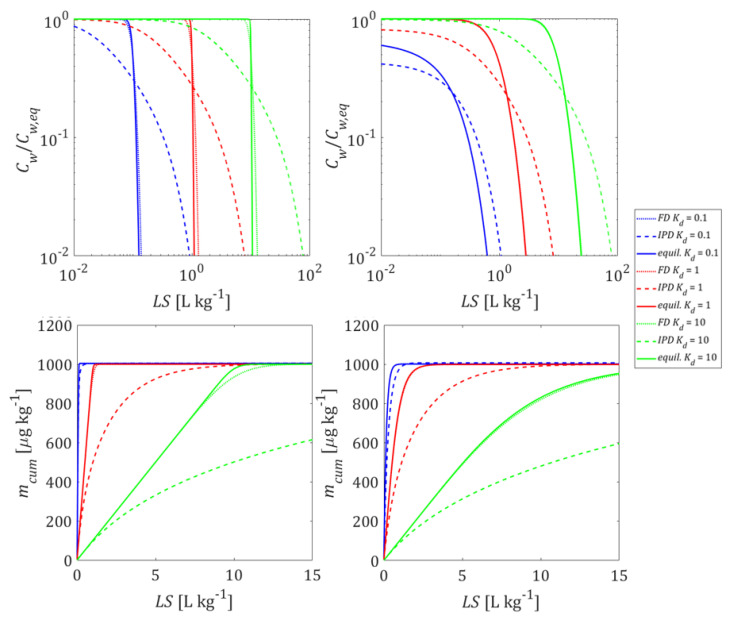
Normalized concentrations ($C_{w}/C_{w,eq}$) as well as cumulative concentrations ($m_{cum}$ ) in the column effluent vs. time (expressed as liquid to solid ratio: *LS*) for different mass-transfer processes, given the initial conditions depicted in Figure 6. **Left column**: without dispersion; **Right column**: with dispersion.

Figure 7 shows concentrations in aqueous leachates that correspond to the initial conditions shown in Figure 6. If leaching is limited by IPD, then the leaching process is slower and concentrations at early times are considerably lower than in the FD or the equilibrium model. This is due to the retarded diffusive transport within the tortuous intragranular pores and the correspondingly small effective diffusion coefficients ($D_{e}$). The contaminant release rate becomes lower and lower over time. Leachate concentrations decrease first with the square root of time (typical for transient diffusion) and then exponentially (see Figure 7 without dispersion, and Appendix D for details about the development of the internal mass transfer resistance over time). Note, the cumulative concentration curves confirm the mass conservation of the numerical solutions.

### 3.3. Nonlinear Sorption Isotherms 

For many of the environmental contaminants and solid materials that are typically analyzed in column leaching tests, non-linear sorption isotherms describe the distribution of solutes between the aqueous and solid phases. The significance of this non-linearity for the development of the conditions in the column before the leaching starts has been analyzed exemplarily by defining Freundlich isotherms that result in the same “effective” $K_{d}$ for the aqueous concentration at equilibrium: $K_{fr}=K_{d}/C_{w,eq}^{\frac{1}{n}-1}$.

**Figure 8:**
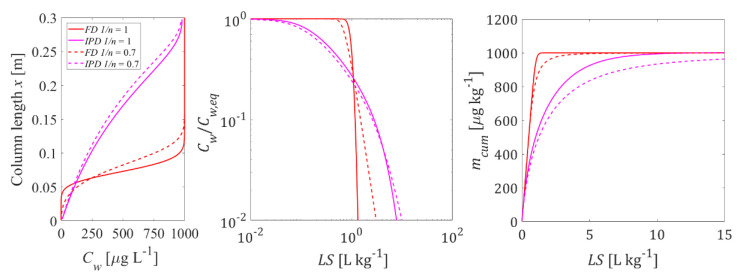
Influence of sorption non-linearity: initial aqueous concentration distribution in the column after the first flooding (**left graph**) and column effluent concentration (normalized: **mid graph**, cumulative: **right graph**) vs. time (expressed as liquid to solid ratio: *LS*); solid lines: linear sorption ($K_{d}$ = 1 L kg^−1^); dashed lines: non-linear sorption case (Freundlich coefficient $K_{fr}$ = 7.94, exponent 1/n = 0.7); *n* = 0.45, *v* = 1.67 × 10^−5^ m s^−1^, α/*x* = 0 or 0.1, $C_{s,ini}$ = 1000 μg kg^−1^, $t_{c}$ = 5 h, $D_{aq}$ = 1 × 10^−9^ m^2^ s^−1^, ε = 0.05, $d_{p,coarse}$ = 2000 μm.

Figure 8 shows the influence of nonlinear sorption on both the initial concentrations in the column and the leaching curves for the example of $K_{d}\;$= 1 L kg^−1^ when no dispersion is considered. The differences in the concentration distribution before percolation starts are moderate. Concentration profiles tend to be smoother with nonlinear sorption with a slightly lower maximum concentration at the column outlet for low to mid $K_{d}$ values if dispersion is considered (see SM, Figure S1). Differences become more obvious in the tailing part of the leaching curves. Freundlich exponents smaller than 1 result in a longer tailing as is expected. The effect of nonlinear sorption looks similar to the dispersion effect, in both cases the leaching curves show more tailing (see SM, Figure S2). Nonlinearity of sorption is notably less significant than kinetic limitations in the mass transfer mechanisms.

### 3.4. Impact of Heterogeneous Sample Composition 

Real world materials that are typically investigated in leaching tests are not always homogeneous. Although the sample might be addressed as ‘one material’, its individual grains have different sizes and differ most likely also in other properties such as porosity, tortuosity, sorption capacity, etc., and may contain different amounts of the contaminants of interest. In order to illustrate the impact of material heterogeneity, we have carried out several numerical leaching experiments with hypothetical material compositions.

**Figure 9:**
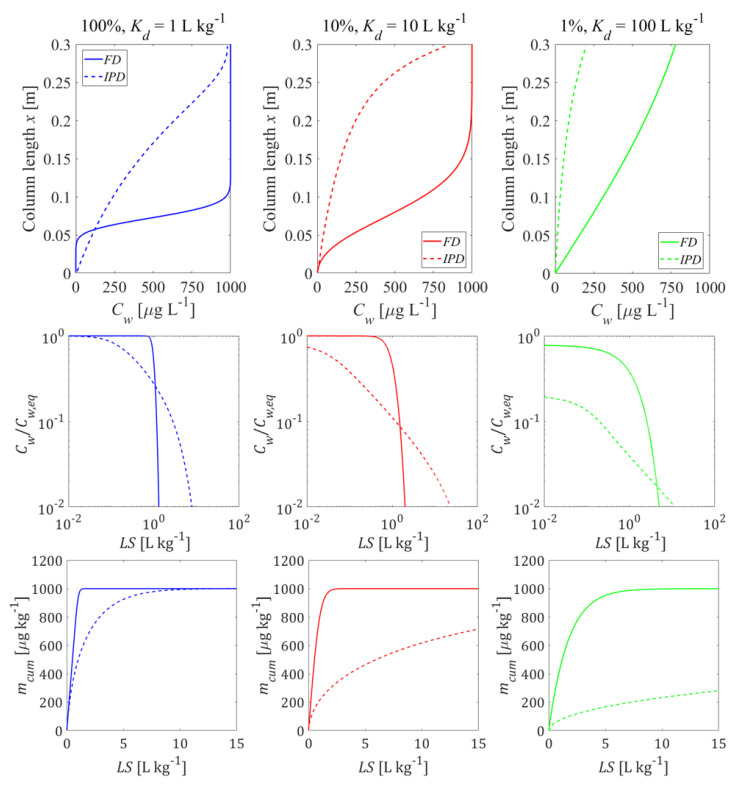
Behavior of bi-modal material compositions of sorbing and non-sorbing particles: initial concentration distribution in the column after the first flooding (**top panel**) and column effluent concentration (normalized: **mid panel**, cumulative: **bottom panel**) vs. time (expressed as liquid to solid ratio: LS). Left column: homogeneous case with average $K_{d}\;$(= $K_{d,av}\;$ = 1 L kg^−1^); mid column: only 10% of the particles carry the contaminant at $K_{d}\;$ =$\;10\times K_{d,av}$; right column: only 1% of the particles carry the contaminant at $K_{d}$ = $100\times K_{d,av}$; the average $K_{d,av}$ of the entire material is the same for all compositions; solid lines: film diffusion cases, dashed lines: intraparticle diffusion case; *n* = 0.45, *v* = 1.67 × 10^−5^ m s^−1^, α = 0 (no dispersion), $C_{s,ini}$ = 1000 μg kg^−1^, $t_{c}$ = 5 h, $D_{aq}$ = 1 × 10^−9^ m^2^ s^−1^, ε = 0.05,$\;d_{p,coarse}$ = 2000 μm.

First, we consider three bi-modal material compositions. Each of these compositions consist of a fraction of contaminated particles (e.g., particulate organic carbon particles with high $K_{d}$) and another fraction of particles that neither contain contaminants nor possess any sorption capacity. If equilibrium conditions prevail during the first flooding and leaching, the heterogeneity of the sample does not matter, it is simply the average $K_{d}$ ($K_{d,av}$) that rules. The situation changes if mass transfer between the solid and the aqueous phases is limited due to diffusion (FD or IPD). If only a small fraction of the particles in the sample carries the compounds of interest, the volume of particles released by the compound and thus the surface area available for mass transfer becomes much smaller. This may lead to pronounced non-equilibrium conditions after first flooding (see, e.g., Equations (A26) and (A30)) and during leaching. Figure 9 shows a comparison of the initial concentration profiles in the column after the first flooding, as well as the corresponding leaching curves that would develop for the three bi-modal material compositions (100/10/1% of the material is contaminated at a $K_{d}=1/10/100$ L kg^−^^1^, respectively). A small fraction of strong sorbents showed lower desorption rates compared to a large fraction of the weak sorbents. For this “exotic” case where only 1% of the particles carries all the contamination, initial nonequilibrium and long tailing was observed. This effect was very pronounced for intraparticle pore diffusion; the concentrations initially started on a plateau (“like equilibrium”), but then rapidly declined and showed a pronounced tailing and decrease with the square root of time (or *LS*). It may be noted, that longitudinal dispersion becomes less relevant if non-equilibrium conditions prevail at high $K_{d}$ values (see Figures S3 and S4 in SM). If such pronounced initial nonequilibrium is observed, then extended periods of time would be needed to equilibrate the water in the column with the solids (e.g., a manifold of the contact time of 5 h).

Samples consisting of mixtures of different particle sizes represent another typical and frequently occurring case of material heterogeneity. To illustrate the impact of such grain size heterogeneity, two bi-modal grain size distributions are considered here, introducing two grain sizes, coarse particles having a diameter of $d_{p,coarse}=$ 2000 μm, and fine particles with $d_{p,fine}=$ 63 μm. The 1st hypothetical grain size distribution consists of 10% fine particles and 90% coarse particles, the 2nd distribution of 90% fine particles and 10% coarse particles.

**Figure 10:**
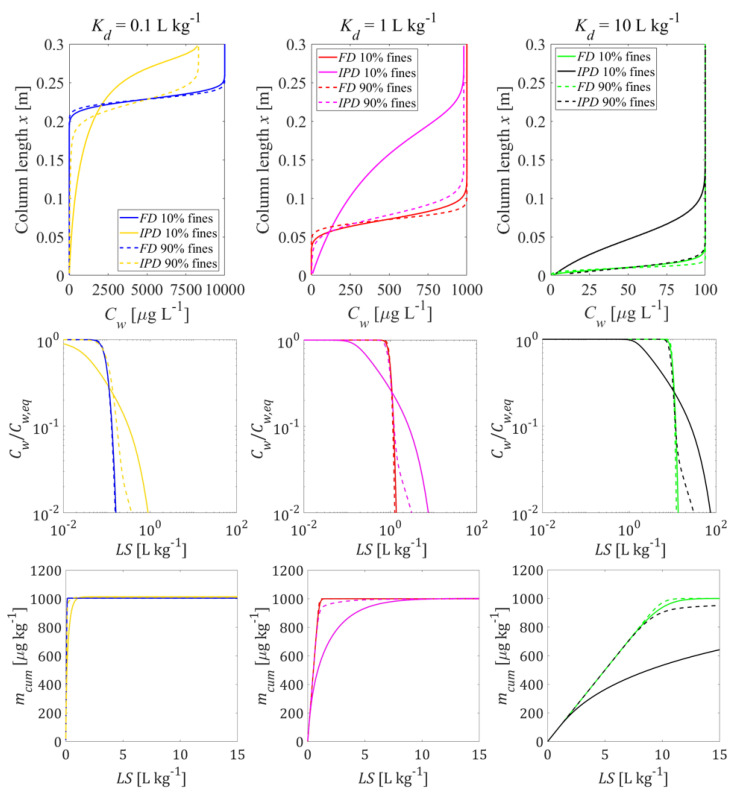
Behavior of the bi-modal material compositions of fine and coarse particles: initial concentration distribution in the column after the first flooding (**top panel**) and column effluent concentration (normalized: **mid panel**, cumulative: **bottom panel**) vs. time (expressed as liquid to solid ratio: *LS*); solid lines: fine particle mass fraction 10%; dashed lines: fine particle mass fraction 90%. (*n* = 0.45, *v* = 1.67 × 10^−5^ m s^−1^, α = 0 (no dispersion), $C_{s,ini}$ = 1000 μg kg^−1^, $t_{c}$ = 5 h, $D_{aq}$ = 1 × 10^−9^ m^2^ s^−1^, ε = 0.05,$\;d_{p,coarse}$ = 2000 μm, $d_{p,fine}$ = 63 μm).

If mass transfer is limited by film diffusion, the establishment of the initial conditions as well as leaching (Figure 10) occurs under conditions close to equilibrium for both grain size distributions at all $K_{d}$ values (0.1, 1, and 10 L kg^−1^). While the shapes of all leaching curves are very similar, their locations are shifted in time according to the different $K_{d}$ values by a factor of 10. If intraparticle pore diffusion is considered, tailing is observed if coarse particles predominate. This applies to both, the development of initial conditions in the column and leaching. If fine particles predominate, the leaching is close to equilibrium at early times; at later times, tailing is observed with the typical square root of time behavior. Considering the dispersion effect, non-equilibrium concentrations can be seen at the column effluent after first flooding especially at low $K_{d}$ values ($K_{d}=$0.1 L kg^−1^). Initial non-equilibrium conditions become more salient for intraparticle pore diffusion if coarse particles predominate (see Figures S5 and S6 in SM).

**Figure 11:**
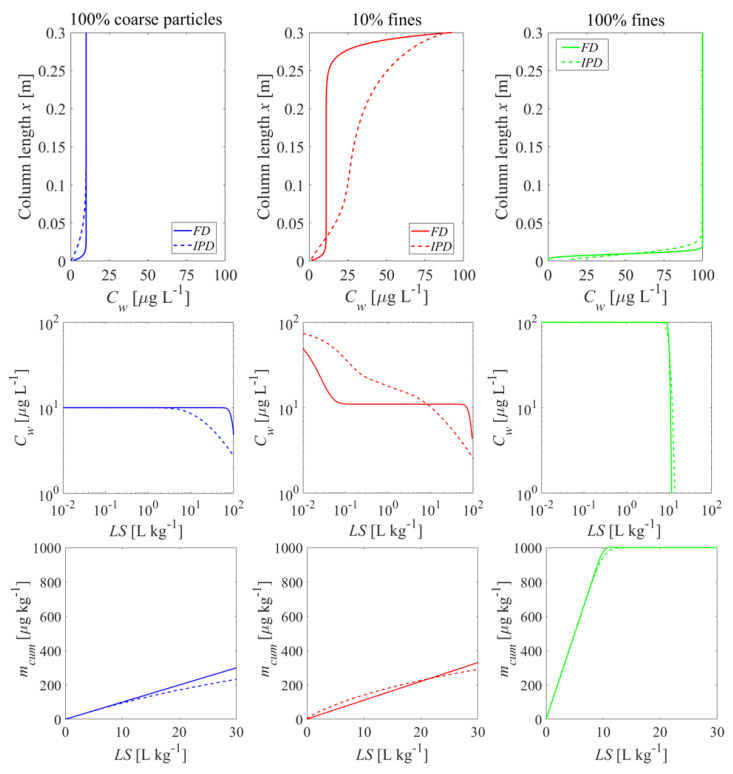
Behavior of bi-modal material compositions of fine particles with low sorption capacity ($K_{d}=$10 L kg^−^^1^) and coarse particles with high sorption capacity ($K_{d}=$ 100 L kg^−^^1^): initial concentration distributions in the column after the first flooding (**top panel**) and the column effluent concentration (normalized: **mid panel**, cumulative: **bottom panel**) vs. time (expressed as liquid to solid ratio: *LS*). Left column: 100% coarse particles; mid column: mixed sample with 10% fine particles; right column: 100% fine particles; *n* = 0.45, *v* = 1.67 × 10^−5^ m s^−1^, α = 0 (no dispersion), $C_{s,ini}$ = 1000 μg kg^−1^, $t_{c}$ = 5 h, $D_{aq}$ = 1 × 10^−9^ m^2^ s^−1^, ε = 0.05,$\;d_{p,coarse}\;$ = 2000 μm, $d_{p,fine}$ = 63 μm.

Combining the heterogeneity of particle size (d) and sorption capacity ($K_{d}$), we consider three material compositions in the third case, which aims at showing circumstances where strong non-equilibrium conditions may be expected. For many materials this is probably not very realistic, but it may occur in material mixtures where a small particle fraction carries a “labile” contamination with a low $K_{d}$ vs. just a few large particles carrying the contaminant with a large $K_{d}$. A hypothetical mixture containing 10% of fine particles with low sorption capacity ($K_{d}=$ 10 L kg^−^^1^) and 90% of coarse particles with high sorption capacity ($K_{d}=$ 100 L kg^−^^1^) is compared with two extreme cases where a hypothetical sample only contains pure fine particles with low sorption capacity and another hypothetical sample contains pure coarse particles with high sorption capacity. Figure 11 shows the initial concentration distribution for these three compositions after the first flooding period as well as the corresponding leaching curves. Sorption equilibrium is achieved rapidly if the sample consists of only fine particles with a small $K_{d}$ or only coarse particles with a high $K_{d}$. Pure coarse material with a high $K_{d}$ shows a low equilibrium concentration ($C_{w,eq}=C_{s,ini}/K_{d}=$ 1000 μg kg^−^^1^/100 L kg^−^^1^ = 10 μg L^−^^1^) while pure fine material with a low $K_{d}$ presents a much higher equilibrium concentration ($C_{w,eq}=C_{s,ini}/K_{d}=$ 1000 μg kg^−^^1^/10 L kg^−^^1^ = 100 μg L^−^^1^) after a short flow distance. Interestingly, the mixed case where 10% of the column is fine material caused a high concentration which would be sorbed by the coarse materials leading to a slightly higher plateau concentration compared to pure coarse materials. The pollutants were redistributed between fine and coarse materials during the first flooding of the column. The concentration increase towards the outlet of the column in the mixed case is due to fast desorption from the fine material followed by slow sorption by the coarse material. The redistribution is almost complete at the inlet of the column because of the long residence time ($t_{c}\;$= 5 h). Since the fine particles make up only to 10% of the total mass, they are already depleted in contaminant concentrations inside the column and in equilibrium with the coarse particles (reflecting both extreme cases). The front of the high concentration caused by the fine particles is already close to the outlet, while the rest is in equilibrium with the 90% coarse particle fraction.

The leaching curve of the mixed case (red lines) reflects the properties of the two pure (homogeneous) cases with either fine or coarse particles. Ten percent of the fine particles with low sorption capacity led to a peak effluent concentration which was only slightly lower than the equilibrium concentration of the pure fine particles with low sorption capacity. However, because the fine particles made up only 10% of the total mass, this peak concentration leached out rapidly and the eluate concentrations followed the coarse particles with a high sorption capacity for long time periods (blue curves). Although this may appear to indicate non-equilibrium conditions (because of the rapid initial decline of the concentrations followed by a plateau or “tailing”), leaching from fine and coarse particles occurs at, or close to equilibrium. Compared to FD, the IPD in the mixed sample showed a slower concentration decline because of the desorption kinetics of the IPD of the coarse particles was slower than the case of FD and on the long-term control release kinetics. For the cumulative mass release the mixed case is close to the coarse material for both the FD and the IPD, whereas the fine-grained particles showed a much higher and faster release (see Figure 11: bottom panel).

## 4. Summary and Conclusions

We conducted numerical simulations to investigate the release characteristics of low to strongly sorbing compounds ($K_{d}=0.1$–100 L kg^−1^) in column leaching tests. Two different scenarios for the establishment of the initial conditions before the start of the leaching phase were considered: a fully pre-equilibrated column and a more realistic scenario where a column is flooded with water from the bottom. In order to highlight the effect of mass transfer limitations, two mechanisms are compared: film diffusion and intra-particle diffusion. Cases without and with dispersion illustrate how dispersive mixing may mask diffusion limited mass transfer. Furthermore, we looked into the impact of heterogeneous sample compositions in terms of reactive particle fractions and particle sizes. Since possible parameter combinations amount to almost infinite numbers, we have limited our analysis to just a few exemplary cases that illustrate the role of individual material properties. These few cases already show that virtually any leaching behavior can be produced with highly heterogeneous samples (depending on the mixing of different materials). The most important conclusions are:

**Initial conditions** have a significant impact on leaching at low $K_{d}$ values ($K_{d}\;$< 1 L kg^−1^). With increasing $K_{d},$ the differences between pre-equilibrium and non-equilibrium conditions gradually vanish for $K_{d}\;$> 10 L kg^−1^ (see Figure 5). Compounds with very low $K_{d}$ (“salts”) would reach extremely high concentrations ($K_{d}\;$<< 1 L kg^−1^) at the column outlet (see Figure 4) potentially leading to enhanced dispersion due to density fingering. The $K_{d}$ values derived from retardation factors ($R_{d}$ in Equation (4)) would be underestimated if the conditions in the column after the first flooding are not appropriately considered, due to a “phase shift” in normalized concentrations curves ($C_{w}/C_{w,eq}$ vs. *LS*).

**Dispersion** generally causes “smoothing” of concentrations gradients in the column and tends to “mask” film and intraparticle diffusion characteristics due to enhanced “mixing” of the solute within the column. It may lead to smaller initial concentrations at the column outlet after the first flooding period than expected for equilibrium; this is pronounced especially at low $K_{d}$ values (see Figure 7 and Figure S2), which may be interpreted as non-equilibrium, but is just a consequence of dilution by dispersive mixing. 

**Intraparticle pore diffusion** (IPD) generally shows slower desorption kinetics than **film diffusion** (FD) through an aqueous boundary layer. This is due to the much smaller effective diffusion coefficient in the intraparticle pores and the large diffusion distance that develops inside the particle over time, resulting in the typical square root of time decrease of concentrations (a slope of 1/2 is observed in log-log plots of leaching curves, see Figure 7, Figure 9 and Figure 10). IPD is more sensitive to the variation of particle sizes than FD (see Figure 10). Mass transfer limitations in an aqueous boundary layer commonly exists for surface adsorbed compounds and easily soluble solids (“salts”). Elements such as heavy metals, which are slowly released from the solid phase, would require much lower solid state diffusion coefficients; if reaction fronts propagate into the particle releasing metals, intraparticle (solid) diffusion models apply again (shrinking core), which are very similar to the IPD approach used here. 

**Non-linear sorption** has little influence on the leaching test results if the “right” effective $K_{d}$ value is calculated for the proper concentration range (since for the nonlinear sorption the $K_{d}$ depends on the concentration, large deviations may occur if just the $K_{fr}$ is determined far away of the sample’s concentration is used as “$K_{d}$”); nevertheless, as concentrations decrease nonlinear sorption causes more tailing (see Figure 8). 

**Heterogeneous samples** with only a small fraction of strongly sorbing particles lead to much slower desorption rates (because of less surface area), especially if mass release is limited by intraparticle pore diffusion (see Figure 9). In extreme cases (just 1% of the material is contaminated at $K_{d}=100\times K_{d,av}$), leaching may start at a plateau (suggesting equilibrium), but far below equilibrium concentrations ($C_{w,peak}\ll C_{w,eq}$) and concentrations later decrease further; The $K_{d}$ values derived from the initial aqueous concentration ($K_{d}=C_{s,ini}/C_{w,peak}$) would be overestimated while the $K_{d}$ values calculated from retardation factors would be underestimated. 

In contrast to that, already relatively small amounts of fine particles lead to initial equilibrium, but long-term tailing occurs and is dominated by the coarse particle fraction, especially for intraparticle pore diffusion. Since our FD simulations are close to equilibrium, results are not very affected by grain size heterogeneity (see Figure 10). Material mixtures of small amounts of fine particles (10%) with low sorption capacity ($K_{d}=$10 L kg^−^^1^) and large amounts of coarse particles with high sorption capacity ($K_{d}=$ 100 L kg^−^^1^), exhibit the respective characteristics of each of the individual components in different time periods (see Figure 11). Small amounts of fine particles with low sorption capacity dominate short term behavior of the mixtures and lead to a peak effluent concentration ($C_{w,peak}$) which approaches the equilibrium concentration expected for fine particles (see Figure 11). Since the mass fraction of fine particles is small (10%), the leachate concentrations drop rapidly and reach slightly higher equilibrium levels of 100% pure coarse particles due to the redistribution of pollutants between fine and coarse particles. Ten percent of fine particles with low sorption capacity causes a high equilibrium concentration which are sorbed by the coarse particles with high sorption capacity. $K_{d}$ values derived from the initial aqueous concentration ($K_{d}=C_{s,ini}/C_{w,peak}$) would be underestimated, while $K_{d}$ values derived from the following plateau concentration would be overestimated. Cumulative mass release, however, is often quite insensitive to mass transfer mechanisms (FD or IPD) especially for *LS* < 5 (see Figure 11).

## Figures and Tables

**Table 1:** Summary of parameter values and ranges used to set up the numerical experiments.

Property	Symbol (Unit)	Reference and [Alternative Values]
Net column length	$X_{col}$ (cm)	30
Inner column diameter	$D_{c}$ (cm)	5.46
Total volume of column	$V_{tot}\;\left(L\right)$	0.70
Dry solid density	$\rho_{s}$ (kg L^−1^)	2.60
Inter-granular porosity	*n* (-)	0.45
Intraparticle porosity	ε (-)	0.05
Solid mass in column	$m_{d}$ (kg)	1
Liquid to solid ratio in column	$LS_{col}\;$(L kg^−1^)	0.31
Initial concentration in solid phase	$C_{s,ini}$ (µg kg^−1^)	1000
Contact time	$t_{c}$ (h)	5
Dispersivity	α (m)	[0, 0.03]
Water flow velocity	*v* (m s^−1^)	1.67 × 10^−5^
Aqueous diffusion coefficient	$D_{aq}$ (m^2^ s^−1^)	1 × 10^−9^
Particle diameters	d (µm)	[63, 2000]
Distribution coefficients	$K_{d}$ (L kg^−1^)	[0.1, 1, 10, 100]
Freundlich coefficients	$K_{fr}$ (µg kg^−^^1^:(µg L^−^^1^)^1/*n*^)	[1.58, 7.94, 39.81]
Freundlich exponent	1/n	0.7
Sherwood number	$Sh=2+0.1Pe^{1/2}$ (-)	[2.1, 2.6]

**Figure A3:**
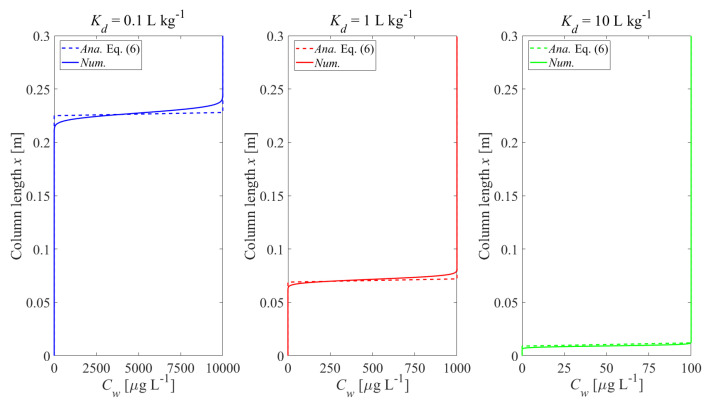
Concentration vs. distance in the up-flow column test after the first flooding of the column (initial condition). Comparison of the analytical solution (Equation (6), dashed lines) and numerical solution (solid lines); *n* = 0.45, *v* = 1.67 × 10^−5^ m s^−1^, α = 0 (no dispersion), $C_{s,ini}$ = 1000 μ g kg^−1^, $t_{c}$ = 5 h, $d_{p,fine}$ = 63 μ m.

**Figure A4:**
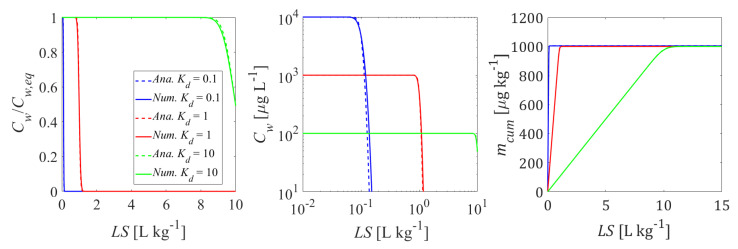
Normalized and absolute concentration ($C_{w}/C_{w,eq},C_{w}$) as well as cumulative concentration ($m_{cum}$ ) in the column effluent vs. time (expressed as liquid to solid ratio: *LS*) for initial conditions shown in Figure A3; comparison of analytical solution (Equation (6), dashed lines) and numerical solution (solid lines).

## Data Availability

The data presented in this study are available in this article.

## Supplementary Materials

The following are available online at https://www.mdpi.com/article/10.3390/ma14164708/s1, Figure S1. Initial concentration profiles in the column after the first flooding (up-flow) without and with dispersion (top and bottom panel); solid lines: linear sorption; dashed lines: non-linear sorption cases (based on a Freundlich exponent 1/n = 0.7), *n* = 0.45, *v* = 1.67 × 10^−^^5^ m s^−^^1^, α/*x* = 0 or 0.1, $C_{s,ini}$ = 1000 μg kg^−^^1^, $t_{c}$ = 5 h, $D_{aq}$ = 1 × 10^−^^9^ m^2^ s^−^^1^, ε = 0.05, $d_{p,coarse}$ = 2000 μm, Figure S2. Normalized concentrations ($C_{w}/C_{w,eq}$) as well as cumulative concentrations ($m_{cum}$) in the column effluent vs. time (expressed as liquid to solid ratio: *LS*) for different initial conditions depicted in Figure S1; solid lines: linear sorption; dashed lines: nonlinear sorption. Left column: without dispersion; right column: with dispersion, Figure S3. Initial concentration distribution in the column after the first flooding (up-flow) for different bi-modal compositions of sorbing and non-sorbing particles; left column: homogeneous case with average $K_{d}$ (=$K_{d,av}\;$= 1 L kg^−^^1^); mid column: only 10% of the particles carry the contaminant at $K_{d}\;$=$\;10\times K_{d,av}$; right column: only 1% of the particles carry the contaminant at $K_{d}$ = $100\times K_{d,av}$; the average $K_{d,av}$ of the entire material is the same for all compositions; solid lines: film diffusion case, dashed lines: intraparticle diffusion case. Top panel: without dispersion; bottom panel: with dispersion; *n* = 0.45, *v* = 1.67 × 10^−^^5^ m s^−^^1^, α/*x* = 0 or 0.1, $C_{s,ini}$ = 1000 μg kg^−^^1^, $t_{c}$ = 5 h, $D_{aq}$ = 1 × 10^−^^9^ m^2^ s^−^^1^, ε = 0.05, $d_{p,coarse}$ = 2000 μm, Figure S4. Normalized concentrations ($C_{w}/C_{w,eq}$) as well as cumulative concentrations ($m_{cum}$) in the column effluent vs. time (expressed as liquid to solid ratio: *LS*) for different combinations of sorbing particles and distribution coefficients (initial conditions depicted in Figure S3); left: without dispersion; right: with dispersion; solid lines: film diffusion cases, dashed lines: intraparticle diffusion cases, Figure S5. Initial concentration distribution in the column after the first flooding (up-flow) for two different bi-modal grain size distributions of fine and coarse particles; solid lines: fine particle mass fraction 10%; dashed lines: fine particle mass fraction 90%. (*n* = 0.45, *v* = 1.67 × 10^−^^5^ m s^−^^1^, α/*x* = 0 or 0.1, $C_{s,ini}$ = 1000 μg kg^−^^1^, $t_{c}$ = 5 h, $D_{aq}$ = 1 × 10^−^^9^ m^2^ s^−^^1^, ε = 0.05,$\;d_{p,coarse}$ = 2000 μm, $d_{p,fine}$ = 63 μm); top panel: without dispersion; bottom panel: with dispersion, Figure S6. Influence of different grain size fractions and distribution coefficients on normalized concentrations ($C_{w}/C_{w,eq}$) as well as cumulative concentrations ($m_{cum}$) in the column effluent vs. time (expressed as liquid to solid ratio *LS*); left: without dispersion; right: with dispersion; solid lines: fine particle mass fraction 10%; dashed lines: fine particle mass fraction 90%; kinetic parameters are the same as Figure S5, Figure S7. Initial concentration distribution in the column after the first flooding (up-flow) for different bi-modal material compositions of fine particles with low sorption capacity ($K_{d}=$ 10 L kg^−1^) and coarse particles with high sorption capacity; left: 100% coarse particles ($K_{d}=$ 100 L kg^−1^); middle: mixed sample with 10% fine particles; right: 100% fine particles; solid lines: film diffusion (FD), dashed lines: intraparticle diffusion cases (IPD); *n* = 0.45, *v* = 1.67 × 10^−^^5^ m s^−^^1^, α/*x* = 0 or 0.1, $C_{s,ini}$ = 1000 μg kg^−^^1^, $t_{c}$ = 5 h, $D_{aq}$ = 1 × 10^−^^9^ m^2^ s^−^^1^, ε = 0.05,$\;d_{p,coarse}\;$= 2000 μm, $d_{p,fine}$ = 63 μm; top panel: without dispersion; bottom panel: with dispersion, Figure S8. Leachate concentrations ($C_{w}$) as well as cumulative concentrations ($m_{cum}$) in the column effluent vs. time (expressed as liquid to solid ratio: *LS*) for different combinations of fine particles with low sorption capacity ($K_{d}=$ 10 L kg^−1^) and coarse particles with high sorption capacity ($K_{d}=$ 100 L kg^−1^); left: without dispersion; right: with dispersion; solid lines: film diffusion cases, dashed lines: intraparticle diffusion cases; kinetic parameters are the same as Figure S7.

## Appendix A. Empirical Relationships for the Estimation of Sherwood Numbers

There are many studies available in the literature in which solid-liquid mass transfer in fluidized beds and flow through systems are investigated over a wide range of Reynolds numbers. Most of these correlations can be adequately described by the following equation:

(A1) $$Sh=A+BRe^{\theta }Sc^{\gamma }$$

where Sh is the Sherwood number. *A* is a constant (theoretically = 2 for spherical particles in a stagnant infinite medium) and *B* is a constant to be determined by regression analysis of experimental data. Re and Sc denote the Reynolds and Schmidt number which are defined as:

(A2) $$Sc=\frac{\eta }{\rho_{L}D_{aq}}\;Re=\frac{\rho_{L}d\;v}{\eta }$$

where η [M L^−^^1^ T^−^^1^] denotes the dynamic viscosity of the fluid. $\rho_{L}$ [M L^−3^] is the density of the fluid and v [L T^−1^] denotes the flow velocity.

The empirical exponents θ and γ in Equation (A1) may be determined experimentally or from theory. The Blasius (1908) solved the Navier-Stokes equation and continuity equation for laminar flow over a sharp leading edge and found that the ratio of fluid velocity boundary layer thickness to concentration boundary layer thickness is proportional to the Schmidt number with a power of 1/3 (=γ in Equation (A1)) which is widely used in literature [37,38,39,40,41,42,43]. Liu et al. (2014) showed a higher empirical exponent γ of 1/2 based on penetration theory [35,44]. θ values depend on the experimental setup and are generally adapted from experimental data. Most of the empirical relationships show that θ values lie in the range of 0.5–0.75 [35,37,39,40,41,42,43].

Liu et al. (2014) proposed an empirical relationship for mass transfer in packed beds only based on the Peclet number (Pe=Re×Sc) [35]:

(A3) $$Sh=2+0.1Pe^{1/2}$$

Equation (A3) is equivalent to Equation (A1) for θ=γ=1/2. This Sherwood number correlation was applied in the numerical models. Sh numbers obtained for the chosen column setup were close to 2 indicating slow mass transfer close to the theoretical limit (2).

## Appendix B. Film Diffusion Coupled to Advective-Dispersive Transport

Equation (10) in the main text shows the governing equations of film diffusion coupled to advective-dispersive transport. These partial differential equations are solved numerically using the finite volume method (as illustrated in Figure A1a).

Discretizing the transport operator in space while keeping the time derivative yields the following system of ordinary differential equations:

(A4) $$\begin{array}{c} \frac{\partial C_{w,j}}{\partial t}=D_{L}\frac{\left(C_{w,j-1}-2C_{w,j}+C_{w,j+1}\right)}{\Delta x^{2}}-v\frac{\left(C_{w,j}-C_{w,j-1}\right)}{\Delta x}\\ +\frac{D_{aq}}{\delta_{f}}\frac{6\left(1-n\right)}{nd}\left(\frac{C_{s,j}}{K_{d}}-C_{w,j}\right)\\ \frac{\partial C_{s,j}}{\partial t}=-\frac{D_{aq}}{\delta_{f}}\frac{\;6}{\rho_{s}\;d\;}\left(\frac{C_{s,j}}{K_{d}}-C_{w,j}\right) \end{array}$$

where $C_{w,j}$ [M L^−^^3^], $C_{w,j-1}$ [M L^−^^3^] and $C_{w,j+1}$ [M L^−^^3^] denote the solute concentration in the water phase in volume j, j−1 and j+1, respectively. $C_{s,j}$ [M M^−^^1^] denotes the solute concentration in the solid phase in volume j.

The approximation of the time derivative of Equation (A4) can be expressed as the concentration difference between the new and the previous time, divided by the time interval Δt. A time weighting factor φ was used to navigate between implicit and explicit time integration. For φ=0.5, the Crank-Nicholson-scheme is realized, whereas for φ=0 and φ=1, the fully implicit and explicit scheme is used, respectively.

(A5) $$\begin{array}{c} \frac{C_{w,j}^{k+1}-C_{w,j}^{k}}{\Delta t}=\left(1-\varphi \right)\left(D_{L}\frac{\left(C_{w,j-1}^{k+1}-2C_{w,j}^{k+1}+C_{w,j+1}^{k+1}\right)}{\Delta x^{2}}-v\frac{\left(C_{w,j}^{k+1}-C_{w,j-1}^{k+1}\right)}{\Delta x}+\frac{D_{aq}}{\delta_{f}}\frac{6\left(1-n\right)}{nd}\left(\frac{C_{s,j}^{k+1}}{K_{d}}-C_{w,j}^{k+1}\right)\right)\\ +\varphi \left(D_{L}\frac{\left(C_{w,j-1}^{k}-2C_{w,j}^{k}+C_{w,j+1}^{k}\right)}{\Delta x^{2}}-v\frac{\left(C_{w,j}^{k}-C_{w,j-1}^{k}\right)}{\Delta x}+\frac{D_{aq}}{\delta_{f}}\frac{6\left(1-n\right)}{nd}\left(\frac{C_{s,j}^{k}}{K_{d}}-C_{w,j}^{k}\right)\right)\\ \frac{C_{s,j}^{k+1}-C_{s,j}^{k}}{\Delta t}=-\left(1-\varphi \right)\left(\frac{D_{aq}}{\delta_{f}}\frac{\;6}{\rho_{s}\;d\;}\left(\frac{C_{s,j}^{k+1}}{K_{d}}-C_{w,j}^{k+1}\right)\right)\\ -\varphi \left(\frac{D_{aq}}{\delta_{f}}\frac{\;6}{\rho_{s}\;d\;}\left(\frac{C_{s,j}^{k}}{K_{d}}-C_{w,j}^{k}\right)\right) \end{array}$$

where the indices k and k+1 denote the corresponding concentration values at the previous time step and at the new time step.

In order to solve this system of equations, we may merge the two concentration vectors into a single one (*C* = [$C_{w};C_{s}$]; with the semicolon being a line delimiter):

(A6) $$C={\left[\begin{array}{c} C_{w,1}\\ C_{s,1}\\ \vdots \\ C_{w,j}\\ C_{s,j}\\ \vdots \\ C_{w,N}\\ C_{s,N} \end{array}\right]}_{2N\times 1}={\left[\begin{array}{c} C_{1}\\ C_{2}\\ \vdots \\ C_{2j-1}\\ C_{2j}\\ \vdots \\ C_{2N-1}\\ C_{2N} \end{array}\right]}_{2N\times 1}$$

with jϵ[1, 2, …, N].

A standard method of solving non-linear ordinary equations is the Newton–Raphson scheme [45]. It is based on linearizing the residual function *f*($C^{k+1}$) at the current guess $C_{guess}^{k+1}$ of $C^{k+1}$. The residual function *f*($C^{k+1}$) is defined as:

(A7) $$\begin{array}{cc} f_{2j-1}=\frac{C_{w,j}^{k+1}-C_{w,j}^{k}}{\Delta t} & \\ & -\left(1-\varphi \right)(D_{L}\frac{\left(C_{w,j-1}^{k+1}-2C_{w,j}^{k+1}+C_{w,j+1}^{k+1}\right)}{\Delta x^{2}}-v\frac{\left(C_{w,j}^{k+1}-C_{w,j-1}^{k+1}\right)}{\Delta x}\\ & +\frac{D_{aq}}{\delta_{f}}\frac{6\left(1-n\right)}{nd}\left(\frac{C_{s,j}^{k+1}}{K_{d}}-C_{w,j}^{k+1}\right))\\ & -\varphi (D_{L}\frac{\left(C_{w,j-1}^{k}-2C_{w,j}^{k}+C_{w,j+1}^{k}\right)}{\Delta x^{2}}-v\frac{\left(C_{w,j}^{k}-C_{w,j-1}^{k}\right)}{\Delta x}\\ & +\frac{D_{aq}}{\delta_{f}}\frac{6\left(1-n\right)}{nd}\left(\frac{C_{s,j}^{k}}{K_{d}}-C_{w,j}^{k}\right))\\ f_{2j}=\frac{C_{s,j}^{k+1}-C_{s,j}^{k}}{\Delta t} & +\left(1-\varphi \right)\left(\frac{D_{aq}}{\delta_{f}}\frac{\;6}{\rho_{s}\;d\;}\left(\frac{C_{s,j}^{k+1}}{K_{d}}-C_{w,j}^{k+1}\right)\right)\\ & +\varphi \left(\frac{D_{aq}}{\delta_{f}}\frac{\;6}{\rho_{s}\;d\;}\left(\frac{C_{s,j}^{k}}{K_{d}}-C_{w,j}^{k}\right)\right) \end{array}$$

The residual function vector can be expressed as:

(A8) $$f\left(C^{k+1}\right)={\left[\begin{array}{c} f_{1}\\ f_{2}\\ \vdots \\ f_{2j-1}\\ f_{2j}\\ \vdots \\ f_{2N-1}\\ f_{2N} \end{array}\right]}_{2N\times 1}$$

The residual function vector becomes a zero vector if $C^{k+1}$ is chosen right and a single step of the Newton–Raphson method can be denoted as:

(A9) $$\begin{array}{c} f\left(C^{k+1}\right)\approx f\left(C_{guess}^{k+1}\right)+\left(C^{k+1}-C_{guess}^{k+1}\right){\left.\frac{\partial f\left(C^{k+1}\right)}{\partial C^{k+1}}\right|}_{C^{k+1}=C_{guess}^{k+1}}\\ C^{k+1}=C_{guess}^{k+1}-\frac{f\left(C_{guess}^{k+1}\right)}{J}=C_{guess}^{k+1}-\frac{f\left(C_{guess}^{k+1}\right)}{{\left.\frac{\partial f\left(C^{k+1}\right)}{\partial C^{k+1}}\right|}_{C^{k+1}=C_{guess}^{k+1}}} \end{array}$$

where J denotes the Jacobian matrix, which is the matrix of derivatives of all values of $f\left(C^{k+1}\right)$ with respective to all values of $C^{k+1}$. The residual $f\left(C^{k+1}\right)$ is reevaluated after updating $C^{k+1}$. If the resulting residual is not sufficiently close to zero, $C_{guess}^{k+1}$ is set to the last solution of $C^{k+1}$ and Equation (A9) is reapplied. In our case, the Jacobian matrix can be derived analytically:

(A10) $$J={\left[\begin{array}{ccccc} \frac{\partial f_{1}}{\partial C_{1}} & \frac{\partial f_{1}}{\partial C_{2}} & \cdots & \frac{\partial f_{1}}{\partial C_{2N-1}} & \frac{\partial f_{1}}{\partial C_{2N}}\\ \frac{\partial f_{2}}{\partial C_{1}} & \frac{\partial f_{2}}{\partial C_{2}} & \ldots & \frac{\partial f_{2}}{\partial C_{2N-1}} & \frac{\partial f_{2}}{\partial C_{2N}}\\ \vdots & \vdots & \cdots & \vdots & \vdots \\ \frac{\partial f_{2N-1}}{\partial C_{1}} & \frac{\partial f_{2N-1}}{\partial C_{2}} & \cdots & \frac{\partial f_{2N-1}}{\partial C_{2N-1}} & \frac{\partial f_{2N-1}}{\partial C_{2N}}\\ \frac{\partial f_{2N}}{\partial C_{1}} & \frac{\partial f_{2N}}{\partial C_{2}} & \cdots & \frac{\partial f_{2N}}{\partial C_{2N-1}} & \frac{\partial f_{2N}}{\partial C_{2N}} \end{array}\right]}_{2N\times 2N}$$

In order to ensure the accuracy of the model, error control was employed at each time step and an error vector ($\Delta C^{k+1}$) was used to monitor the difference between the old and new guess values, which is defined as:

(A11) $$\Delta C^{k+1}={\left[\begin{array}{c} \left|C_{1,\;guess,new}^{k+1}-C_{1,\;guess,old}^{k+1}\right|\\ \left|C_{2,\;guess,new}^{k+1}-C_{2,\;guess,old}^{k+1}\right|\\ \vdots \\ \left|C_{2j-1,\;guess,new}^{k+1}-C_{2j-1,\;guess,old}^{k+1}\right|\\ \left|C_{2j,\;guess,new}^{k+1}-C_{2j,\;guess,old}^{k+1}\right|\\ \vdots \\ \left|C_{2N-1,\;guess,new}^{k+1}-C_{2N-1,\;guess,old}^{k+1}\right|\\ \left|C_{2N,\;guess,new}^{k+1}-C_{2N,\;guess,old}^{k+1}\right| \end{array}\right]}_{2N\times 1}$$

The iteration process stops if the maximum value of error vector $\Delta C_{max}^{k+1}$ is smaller than the tolerable error e (e.g., e = 10^−^^15^).

## Appendix C. Intraparticle Pore Diffusion Coupled to Advective-Dispersive Transport

Intraparticle pore diffusion is widely used to describe the sorptive uptake of pollutants in porous materials such as activated carbon, zeolites and many technical materials. Equations (11) and (17) describe intraparticle diffusion coupled to advective-dispersive transport. The intraparticle diffusion model approximates the solid grains as spherical particles. These spherical particles are discretized into a number of shells of equal volume. Mass transfer between solid and intra-granular water phases is assumed to be fast and local equilibrium is assumed. For sorption, the Freundlich isotherm model is employed for nonlinear and linear (exponent = 1) cases. Figure A1b shows the numerical grain model where the spherical grains are divided into *L* shells. For a specific shell *p* in volume *j* the corresponding difference-equations were used [46]:

(A12) $$\begin{array}{c} \frac{1}{\Delta t}\left(\varepsilon C_{p,j}^{k+1}+\rho_{p}K_{fr}{\left(C_{p,j}^{k+1}\right)}^{\frac{1}{n}}-\varepsilon C_{p,j}^{k}-\rho_{p}K_{fr}{\left(C_{p,j}^{k}\right)}^{\frac{1}{n}}\right)\\ =\frac{D_{e}}{r_{p}^{2}}\frac{1-\varphi }{r_{p+0.5}-r_{p-0.5}}\left(r_{p+0.5}^{2}\frac{C_{p+1,j}^{k+1}-C_{p,j}^{k+1}}{r_{p+1}-r_{p}}-r_{p-0.5}^{2}\frac{C_{p,j}^{k+1}-C_{p-1,j}^{k+1}}{r_{p}-r_{p-1}}\right)\\ +\frac{D_{e}}{r_{p}^{2}}\frac{\varphi }{r_{p+0.5}-r_{p-0.5}}\left(r_{p+0.5}^{2}\frac{C_{p+1,j}^{k}-C_{p,j}^{k}}{r_{p+1}-r_{p}}-r_{p-0.5}^{2}\frac{C_{p,j}^{k}-C_{p-1,j}^{k}}{r_{p}-r_{p-1}}\right) \end{array}$$

where the subscripts p+0.5 and p−0.5 represent the corresponding parameter value between shells (p and p+1) and (p and p−1), respectively. Subscript *j* denotes the corresponding parameter value in volume *j*. Subscripts k and k+1 denote the “old” and “new” time levels.

Based on the boundary conditions (Equations (12) and (13), main text), the innermost shell and the outermost shell are treated specially. The solute concentration in the intra-granular water phase at the new time step $(C_{p,j}^{k+1}$) can be expressed as:

(A13) $$\begin{array}{c} \left[\varepsilon +\rho_{p}K_{fr}{\left(C_{p,j}^{k+1}\right)}^{\frac{1}{n}-1}+\frac{D_{e}\Delta t}{r_{p}^{2}}\frac{1-\varphi }{r_{p+0.5}-r_{p-0.5}}\left(\frac{r_{p+0.5}^{2}}{r_{p+1}-r_{p}}+\frac{r_{p-0.5}^{2}}{r_{p}-r_{p-1}}\right)\right]C_{p,j}^{k+1}\\ =\left[\frac{D_{e}\Delta t}{r_{p}^{2}}\frac{1-\varphi }{r_{p+0.5}-r_{p-0.5}}\frac{r_{p+0.5}^{2}}{r_{p+1}-r_{p}}\right]C_{p+1,j}^{k+1}+\left[\frac{D_{e}\Delta t}{r_{p}^{2}}\frac{1-\varphi }{r_{p+0.5}-r_{p-0.5}}\frac{r_{p-0.5}^{2}}{r_{p}-r_{p-1}}\right]C_{p-1,j}^{k+1}\\ +\left[\frac{D_{e}\Delta t}{r_{p}^{2}}\frac{\varphi }{r_{p+0.5}-r_{p-0.5}}\frac{r_{p+0.5}^{2}}{r_{p+1}-r_{p}}\right]C_{p+1,j}^{k}+\left[\frac{D_{e}\Delta t}{r_{p}^{2}}\frac{\varphi }{r_{p+0.5}-r_{p-0.5}}\frac{r_{p-0.5}^{2}}{r_{p}-r_{p-1}}\right]C_{p-1,j}^{k}\\ +\left[\varepsilon +\rho_{p}K_{fr}{\left(C_{p,j}^{k}\right)}^{\frac{1}{n}-1}-\frac{D_{e}\Delta t}{r_{p}^{2}}\frac{\varphi }{r_{p+0.5}-r_{p-0.5}}\left(\frac{r_{p+0.5}^{2}}{r_{p+1}-r_{p}}+\frac{r_{p-0.5}^{2}}{r_{p}-r_{p-1}}\right)\right]C_{p,j}^{k} \end{array}$$

for shell p=2 to shell p=L−1 and

(A14) $$\begin{array}{c} \frac{1}{\Delta t}\left(\varepsilon C_{1,j}^{k+1}+\rho_{p}K_{fr}{\left(C_{1,j}^{k+1}\right)}^{\frac{1}{n}}-\varepsilon C_{1,j}^{k}-\rho_{p}K_{fr}{\left(C_{1,j}^{k}\right)}^{\frac{1}{n}}\right)\\ =\frac{D_{e}}{r_{1}^{2}}\frac{1-\varphi }{r_{1+0.5}-r_{1-0.5}}\left(r_{1+0.5}^{2}\frac{C_{2,j}^{k+1}-C_{1,j}^{k+1}}{r_{2}-r_{1}}\right)+\frac{D_{e}}{r_{1}^{2}}\frac{\varphi }{r_{1+0.5}-r_{1-0.5}}\left(r_{1+0.5}^{2}\frac{C_{2,j}^{k}-C_{1,j}^{k}}{r_{2}-r_{1}}\right)\\ \mathrm{After}\;\mathrm{transformation}:\\ \left[\varepsilon +\rho_{p}K_{fr}{\left(C_{1,j}^{k+1}\right)}^{\frac{1}{n}-1}+\frac{D_{e}\Delta t}{r_{1}^{2}}\frac{1-\varphi }{r_{1+0.5}-r_{1-0.5}}\frac{r_{1+0.5}^{2}}{r_{2}-r_{1}}\right]C_{1,j}^{k+1}\\ =\left[\frac{D_{e}\Delta t}{r_{1}^{2}}\frac{1-\varphi }{r_{1+0.5}-r_{1-0.5}}\frac{r_{1+0.5}^{2}}{r_{2}-r_{1}}\right]C_{2,j}^{k+1}+\left[\frac{D_{e}\Delta t}{r_{1}^{2}}\frac{\varphi }{r_{1+0.5}-r_{1-0.5}}\frac{r_{1+0.5}^{2}}{r_{2}-r_{1}}\right]C_{2,j}^{k}\\ +\left[\varepsilon +\rho_{p}K_{fr}{\left(C_{1,j}^{k}\right)}^{\frac{1}{n}-1}-\frac{D_{e}\Delta t}{r_{1}^{2}}\frac{\varphi }{r_{1+0.5}-r_{1-0.5}}\frac{r_{1+0.5}^{2}}{r_{2}-r_{1}}\right]C_{1,j}^{k} \end{array}$$

for shell 1 (or the innermost shell, p=1) and:

(A15) $$\begin{array}{c} \frac{1}{\Delta t}\left(\varepsilon C_{L,j}^{k+1}+\rho_{p}K_{fr}{\left(C_{L,j}^{k+1}\right)}^{\frac{1}{n}}-\varepsilon C_{L,j}^{k}-\rho_{p}K_{fr}{\left(C_{L,j}^{k}\right)}^{\frac{1}{n}}\right)\\ =\frac{D_{e}}{r_{L}^{2}}\frac{1-\varphi }{R-r_{L-0.5}}\left(R^{2}\frac{C_{w,j}^{k+1}-C_{L,j}^{k+1}}{R-r_{L}}-r_{L-0.5}^{2}\frac{C_{L,j}^{k+1}-C_{L-1,j}^{k+1}}{r_{L}-r_{L-1}}\right)\\ +\frac{D_{e}}{r_{L}^{2}}\frac{\varphi }{R-r_{L-0.5}}\left(R^{2}\frac{C_{w,j}^{k}-C_{L,j}^{k}}{R-r_{L}}-r_{L-0.5}^{2}\frac{C_{L,j}^{k}-C_{L-1,j}^{k}}{r_{L}-r_{L-1}}\right)\\ \mathrm{After}\;\mathrm{transformation}:\\ \left[\varepsilon +\rho_{p}K_{fr}{\left(C_{L,j}^{k+1}\right)}^{\frac{1}{n}-1}+\frac{D_{e}\Delta t}{r_{L}^{2}}\frac{1-\varphi }{R-r_{L-0.5}}\left(\frac{R^{2}}{R-r_{L}}+\frac{r_{L-0.5}^{2}}{r_{L}-r_{L-1}}\right)\right]C_{L,j}^{k+1}\\ =\left[\frac{D_{e}\Delta t}{r_{L}^{2}}\frac{1-\varphi }{R-r_{L-0.5}}\frac{R^{2}}{R-r_{L}}\right]C_{w,j}^{k+1}+\left[\frac{D_{e}\Delta t}{r_{L}^{2}}\frac{1-\varphi }{R-r_{L-0.5}}\frac{r_{L-0.5}^{2}}{r_{L}-r_{L-1}}\right]C_{L-1,j}^{k+1}\\ +\left[\frac{D_{e}\Delta t}{r_{L}^{2}}\frac{\varphi }{R-r_{L-0.5}}\frac{R^{2}}{R-r_{L}}\right]C_{w,j}^{k}+\left[\frac{D_{e}\Delta t}{r_{L}^{2}}\frac{\varphi }{R-r_{L-0.5}}\frac{r_{L-0.5}^{2}}{r_{L}-r_{L-1}}\right]C_{L-1,j}^{k}\\ +\left[\varepsilon +\rho_{p}K_{fr}{\left(C_{L,j}^{k}\right)}^{\frac{1}{n}-1}-\frac{D_{e}\Delta t}{r_{L}^{2}}\frac{\varphi }{R-r_{L-0.5}}\left(\frac{R^{2}}{R-r_{L}}+\frac{r_{L-0.5}^{2}}{r_{L}-r_{L-1}}\right)\right]C_{L,j}^{k} \end{array}$$

for shell *L* (or the outermost shell, p=L).

Based on the mass balance, solute mass change in the external water phase ($M_{w}$) equals the solute mass change in the spherical particles; for better understanding, the simple case of particles with uniform size is shown:

(A16) $$\frac{\partial M_{w}}{\partial t}=V_{w}\frac{\partial C_{w}}{\partial t}=4\pi R^{2}FN_{p}$$

where *F* [M L^−2^ T^−1^] denotes the solute flux density into the external water phase. *R* and $N_{p}$ denote the radius and the total number of the spherical particles. The latter can be calculated by:

(A17) $$N_{p}=\frac{m_{d}}{\rho_{p}\left(\frac{4}{3}\pi R^{3}\right)}$$

The solute flux density into the external water phase is given by:

(A18) $$F=D_{e}\frac{C_{L}-C_{w}}{R-r_{L}}$$

Substituting *F* and $N_{p}$ with Equations (A18) and (A17) in Equation (A16) and taking advection and dispersion into account, the solute concentration in the external water phase at the new time step $C_{w,j}^{k+1}$ can be expressed by:

(A19) $$\begin{array}{l} \begin{array}{cc} \frac{C_{w,j}^{k+1}-C_{w,j}^{k}}{\Delta t} & =\left(1-\varphi \right)(D_{L}\frac{\left(C_{w,j-1}^{k+1}-2C_{w,j}^{k+1}+C_{w,j+1}^{k+1}\right)}{\Delta x^{2}}-v\frac{\left(C_{w,j}^{k+1}-C_{w,j-1}^{k+1}\right)}{\Delta x}\\ & +\frac{3D_{e}m_{d}}{\rho_{p}V_{w}R}\left(\frac{C_{L,j}^{k+1}-C_{w,j}^{k+1}}{R-r_{L}}\right))\\ & +\varphi (D_{L}\frac{\left(C_{w,j-1}^{k}-2C_{w,j}^{k}+C_{w,j+1}^{k}\right)}{\Delta x^{2}}-v\frac{\left(C_{w,j}^{k}-C_{w,j-1}^{k}\right)}{\Delta x}\\ & +\frac{3D_{e}m_{d}}{\rho_{p}V_{w}R}\left(\frac{C_{L,j}^{k}-C_{w,j}^{k}}{R-r_{L}}\right)) \end{array}\\ \begin{array}{c} \mathrm{After}\;\mathrm{transformation}: \end{array}\\ \begin{array}{cc} {}[1+\left(1-\varphi \right) & \left(\frac{3D_{e}m_{d}\Delta t}{\rho_{p}V_{w}R\left(R-r_{L}\right)}+\frac{2D_{L}\Delta t}{\Delta x^{2}}+\frac{v\Delta t}{\Delta x}\right)]C_{w,j}^{k+1}\\ & =\left[\left(1-\varphi \right)\left(\frac{D_{L}\Delta t}{\Delta x^{2}}+\frac{v\Delta t}{\Delta x}\right)\right]C_{w,j-1}^{k+1}+\left[\frac{\left(1-\varphi \right)D_{L}\Delta t}{\Delta x^{2}}\right]C_{w,j+1}^{k+1}\\ & +\left[\frac{\left(1-\varphi \right)3D_{e}m_{d}\Delta t}{\rho_{p}V_{w}R\left(R-r_{L}\right)}\right]C_{L,j}^{k+1}+\left[\varphi \left(\frac{D_{L}\Delta t}{\Delta x^{2}}+\frac{v\Delta t}{\Delta x}\right)\right]C_{w,j-1}^{k}\\ & +\left[\frac{\varphi D_{L}\Delta t}{\Delta x^{2}}\right]C_{w,j+1}^{k}+\left[\frac{\varphi 3D_{e}m_{d}\Delta t}{\rho_{p}V_{w}R\left(R-r_{L}\right)}\right]C_{L,j}^{k}\\ & +\left[1-\varphi \left(\frac{3D_{e}m_{d}\Delta t}{\rho_{p}V_{w}R\left(R-r_{L}\right)}+\frac{2D_{L}\Delta t}{\Delta x^{2}}+\frac{v\Delta t}{\Delta x}\right)\right]C_{w,j}^{k} \end{array} \end{array}$$

In order to solve this system of equations, we may merge the two concentration vectors to a single one (*C* = [$C_{w};C_{p}$]; with the semicolon being a line delimiter):

(A20) $$C={\left[\begin{array}{c} C_{w,1}\\ C_{1,1}\\ \vdots \\ C_{p,1}\\ \vdots \\ C_{L,1}\\ \vdots \\ C_{w,j}\\ C_{1,j}\\ \vdots \\ C_{p,j}\\ \vdots \\ C_{L,j}\\ \vdots \\ C_{w,N}\\ c_{1,N}\\ \vdots \\ C_{p,N}\\ \vdots \\ C_{L,N} \end{array}\right]}_{\left(L+1\right)*N\times 1}={\left[\begin{array}{c} C_{1}\\ C_{2}\\ \vdots \\ C_{p+1}\\ \vdots \\ C_{L+1}\\ \vdots \\ C_{\left(L+1\right)*\left(j-1\right)+1}\\ C_{\left(L+1\right)*\left(j-1\right)+2}\\ \vdots \\ C_{\left(L+1\right)*\left(j-1\right)+p+1}\\ \vdots \\ C_{\left(L+1\right)*j}\\ \vdots \\ C_{\left(L+1\right)*\left(N-1\right)+1}\\ C_{\left(L+1\right)*\left(N-1\right)+2}\\ \vdots \\ C_{\left(L+1\right)*\left(N-1\right)+p+1}\\ \vdots \\ C_{\left(L+1\right)*N} \end{array}\right]}_{\left(L+1\right)*N\times 1}$$

with pϵ[1, 2, …, *L*] and jϵ[1, 2, …, *N*].

Using the Newton–Raphson scheme, the following residual function *f*($C^{k+1}$) is linearized at the current guess $C_{guess}^{k+1}$ of $C^{k+1}$ [45]:

(A21) $$\begin{array}{c} \begin{array}{cc} f_{\left(L+1\right)*\left(j-1\right)+1}= & \left[1+\left(1-\varphi \right)\left(\frac{3D_{e}m_{d}\Delta t}{\rho_{p}V_{w}R\left(R-r_{L}\right)}+\frac{2D_{L}\Delta t}{\Delta x^{2}}+\frac{v\Delta t}{\Delta x}\right)\right]C_{w,j}^{k+1}\\ & -\left[\left(1-\varphi \right)\left(\frac{D_{L}\Delta t}{\Delta x^{2}}+\frac{v\Delta t}{\Delta x}\right)\right]C_{w,j-1}^{k+1}+\left[\frac{\left(1-\varphi \right)D_{L}\Delta t}{\Delta x^{2}}\right]C_{w,j+1}^{k+1}\\ & -\left[\frac{\left(1-\varphi \right)3D_{e}m_{d}\Delta t}{\rho_{p}V_{w}R\left(R-r_{L}\right)}\right]C_{L,j}^{k+1}-\left[\varphi \left(\frac{D_{L}\Delta t}{\Delta x^{2}}+\frac{v\Delta t}{\Delta x}\right)\right]C_{w,j-1}^{k}\\ & -\left[\frac{\varphi D_{L}\Delta t}{\Delta x^{2}}\right]C_{w,j+1}^{k}-\left[\frac{\varphi 3D_{e}m_{d}\Delta t}{\rho_{p}V_{w}R\left(R-r_{L}\right)}\right]C_{L,j}^{k}\\ & -\left[1-\varphi \left(\frac{3D_{e}m_{d}\Delta t}{\rho_{p}V_{w}R\left(R-r_{L}\right)}+\frac{2D_{L}\Delta t}{\Delta x^{2}}+\frac{v\Delta t}{\Delta x}\right)\right]C_{w,j}^{k} \end{array}\\ \begin{array}{c} f_{\left(L+1\right)*\left(j-1\right)+2}=\left[\varepsilon +\rho_{p}K_{fr}{\left(C_{1,j}^{k+1}\right)}^{\frac{1}{n}-1}+\frac{D_{e}\Delta t}{r_{1}^{2}}\frac{1-\varphi }{r_{1+0.5}-r_{1-0.5}}\frac{r_{1+0.5}^{2}}{r_{2}-r_{1}}\right]C_{1,j}^{k+1}\\ -\left[\frac{D_{e}\Delta t}{r_{1}^{2}}\frac{1-\varphi }{r_{1+0.5}-r_{1-0.5}}\frac{r_{1+0.5}^{2}}{r_{2}-r_{1}}\right]C_{2,j}^{k+1}-\left[\frac{D_{e}\Delta t}{r_{1}^{2}}\frac{\varphi }{r_{1+0.5}-r_{1-0.5}}\frac{r_{1+0.5}^{2}}{r_{2}-r_{1}}\right]C_{2,j}^{k}\\ -\left[\varepsilon +\rho_{p}K_{fr}{\left(C_{1,j}^{k}\right)}^{\frac{1}{n}-1}-\frac{D_{e}\Delta t}{r_{1}^{2}}\frac{\varphi }{r_{1+0.5}-r_{1-0.5}}\frac{r_{1+0.5}^{2}}{r_{2}-r_{1}}\right]C_{1,j}^{k}\\ \begin{array}{cc} f_{\left(L+1\right)*\left(j-1\right)+p+1}= & [\varepsilon +\rho_{p}K_{fr}{\left(C_{p,j}^{k+1}\right)}^{\frac{1}{n}-1}\\ & +\frac{D_{e}\Delta t}{r_{p}^{2}}\frac{1-\varphi }{r_{p+0.5}-r_{p-0.5}}\left(\frac{r_{p+0.5}^{2}}{r_{p+1}-r_{p}}+\frac{r_{p-0.5}^{2}}{r_{p}-r_{p-1}}\right)]C_{p,j}^{k+1} \end{array}\\ -\left[\frac{D_{e}\Delta t}{r_{p}^{2}}\frac{1-\varphi }{r_{p+0.5}-r_{p-0.5}}\frac{r_{p+0.5}^{2}}{r_{p+1}-r_{p}}\right]C_{p+1,j}^{k+1}-\left[\frac{D_{e}\Delta t}{r_{p}^{2}}\frac{1-\varphi }{r_{p+0.5}-r_{p-0.5}}\frac{r_{p-0.5}^{2}}{r_{p}-r_{p-1}}\right]C_{p-1,j}^{k+1}\\ -\left[\frac{D_{e}\Delta t}{r_{p}^{2}}\frac{\varphi }{r_{p+0.5}-r_{p-0.5}}\frac{r_{p+0.5}^{2}}{r_{p+1}-r_{p}}\right]C_{p+1,j}^{k}-\left[\frac{D_{e}\Delta t}{r_{p}^{2}}\frac{\varphi }{r_{p+0.5}-r_{p-0.5}}\frac{r_{p-0.5}^{2}}{r_{p}-r_{p-1}}\right]C_{p-1,j}^{k}\\ -\left[\varepsilon +\rho_{p}K_{fr}{\left(C_{p,j}^{k}\right)}^{\frac{1}{n}-1}-\frac{D_{e}\Delta t}{r_{p}^{2}}\frac{\varphi }{r_{p+0.5}-r_{p-0.5}}\left(\frac{r_{p+0.5}^{2}}{r_{p+1}-r_{p}}+\frac{r_{p-0.5}^{2}}{r_{p}-r_{p-1}}\right)\right]C_{p,j}^{k}\\ f_{\left(L+1\right)*j}=\left[\varepsilon +\rho_{p}K_{fr}{\left(C_{L,j}^{k+1}\right)}^{\frac{1}{n}-1}+\frac{D_{e}\Delta t}{r_{L}^{2}}\frac{1-\varphi }{R-r_{L-0.5}}\left(\frac{R^{2}}{R-r_{L}}+\frac{r_{L-0.5}^{2}}{r_{L}-r_{L-1}}\right)\right]C_{L,j}^{k+1}\\ -\left[\frac{D_{e}\Delta t}{r_{L}^{2}}\frac{1-\varphi }{R-r_{L-0.5}}\frac{R^{2}}{R-r_{L}}\right]C_{w,j}^{k+1}-\left[\frac{D_{e}\Delta t}{r_{L}^{2}}\frac{1-\varphi }{R-r_{L-0.5}}\frac{r_{L-0.5}^{2}}{r_{L}-r_{L-1}}\right]C_{L-1,j}^{k+1}\\ -\left[\frac{D_{e}\Delta t}{r_{L}^{2}}\frac{\varphi }{R-r_{L-0.5}}\frac{R^{2}}{R-r_{L}}\right]C_{w,j}^{k}-\left[\frac{D_{e}\Delta t}{r_{L}^{2}}\frac{\varphi }{R-r_{L-0.5}}\frac{r_{L-0.5}^{2}}{r_{L}-r_{L-1}}\right]C_{L-1,j}^{k}\\ -\left[\varepsilon +\rho_{p}K_{fr}{\left(C_{L,j}^{k}\right)}^{\frac{1}{n}-1}-\frac{D_{e}\Delta t}{r_{L}^{2}}\frac{\varphi }{R-r_{L-0.5}}\left(\frac{R^{2}}{R-r_{L}}+\frac{r_{L-0.5}^{2}}{r_{L}-r_{L-1}}\right)\right]C_{L,j}^{k} \end{array} \end{array}$$

The residual function vector can be organized as:

(A22) $$f\left(C^{k+1}\right)={\left[\begin{array}{c} f_{1}\\ f_{2}\\ \vdots \\ f_{p+1}\\ \vdots \\ f_{L+1}\\ \vdots \\ f_{\left(L+1\right)*\left(j-1\right)+1}\\ f_{\left(L+1\right)*\left(j-1\right)+2}\\ \vdots \\ f_{\left(L+1\right)*\left(j-1\right)+p+1}\\ \vdots \\ f_{\left(L+1\right)*j}\\ \vdots \\ f_{\left(L+1\right)*\left(N-1\right)+1}\\ f_{\left(L+1\right)*\left(N-1\right)+2}\\ \vdots \\ f_{\left(L+1\right)*\left(N-1\right)+p+1}\\ \vdots \\ f_{\left(L+1\right)*N} \end{array}\right]}_{\left(L+1\right)*N\times 1}$$

Similar to the film diffusion case (see Appendix B), the $C^{k+1}$ vector can be determined by Equation (A9) as well and the Jacobian matrix of intraparticle pore diffusion case can be expressed as:

(A23) $$J={\left[\begin{array}{ccccc} \frac{\partial f_{1}}{\partial C_{1}} & \frac{\partial f_{1}}{\partial C_{2}} & \cdots & \frac{\partial f_{1}}{\partial C_{\left(L+1\right)*N-1}} & \frac{\partial f_{1}}{\partial C_{\left(L+1\right)*N}}\\ \frac{\partial f_{2}}{\partial C_{1}} & \frac{\partial f_{2}}{\partial C_{2}} & \ldots & \frac{\partial f_{2}}{\partial C_{\left(L+1\right)*N-1}} & \frac{\partial f_{2}}{\partial C_{\left(L+1\right)*N}}\\ \vdots & \vdots & \cdots & \vdots & \vdots \\ \frac{\partial f_{\left(L+1\right)*N-1}}{\partial C_{1}} & \frac{\partial f_{\left(L+1\right)*N-1}}{\partial C_{2}} & \cdots & \frac{\partial f_{\left(L+1\right)*N-1}}{\partial C_{\left(L+1\right)*N-1}} & \frac{\partial f_{\left(L+1\right)*N-1}}{\partial C_{\left(L+1\right)*N}}\\ \frac{\partial f_{\left(L+1\right)*N}}{\partial C_{1}} & \frac{\partial f_{\left(L+1\right)*N}}{\partial C_{2}} & \cdots & \frac{\partial f_{\left(L+1\right)*N}}{\partial C_{\left(L+1\right)*N-1}} & \frac{\partial f_{\left(L+1\right)*N}}{\partial C_{\left(L+1\right)*N}} \end{array}\right]}_{\left(L+1\right)*N\times \left(L+1\right)*N}$$

In order to ensure the accuracy of the model, an error vector ($\Delta C^{k+1}$) was used as described above for Equation (A11):

(A24) $$\Delta C^{k+1}={\left[\begin{array}{c} \left|C_{1,guess,new}^{k+1}-C_{1,guess,old}^{k+1}\right|\\ \left|C_{2,guess,new}^{k+1}-C_{2,guess,old}^{k+1}\right|\\ \vdots \\ \left|C_{p+1,guess,new}^{k+1}-C_{p+1,guess,old}^{k+1}\right|\\ \vdots \\ \left|C_{L+1,guess,new}^{k+1}-C_{L+1,guess,old}^{k+1}\right|\\ \vdots \\ \left|C_{\left(L+1\right)*\left(j-1\right)+1,guess,new}^{k+1}-C_{\left(L+1\right)*\left(j-1\right)+1,guess,old}^{k+1}\right|\\ \left|C_{\left(L+1\right)*\left(j-1\right)+2,guess,new}^{k+1}-C_{\left(L+1\right)*\left(j-1\right)+2,guess,old}^{k+1}\right|\\ \vdots \\ \left|C_{\left(L+1\right)*\left(j-1\right)+p+1,guess,new}^{k+1}-C_{\left(L+1\right)*\left(j-1\right)+p+1,guess,old}^{k+1}\right|\\ \vdots \\ \left|C_{\left(L+1\right)*j,guess,new}^{k+1}-C_{\left(L+1\right)*j,guess,old}^{k+1}\right|\\ \vdots \\ \left|C_{\left(L+1\right)*\left(N-1\right)+1,guess,new}^{k+1}-C_{\left(L+1\right)*\left(N-1\right)+1,guess,old}^{k+1}\right|\\ \left|C_{\left(L+1\right)*\left(N-1\right)+2,guess,new}^{k+1}-C_{\left(L+1\right)*\left(N-1\right)+2,guess,old}^{k+1}\right|\\ \vdots \\ \left|C_{\left(L+1\right)*\left(N-1\right)+p+1,guess,new}^{k+1}-C_{\left(L+1\right)*\left(N-1\right)+p+1,guess,old}^{k+1}\right|\\ \vdots \\ \left|C_{\left(L+1\right)*N,guess,new}^{k+1}-C_{\left(L+1\right)*N,guess,old}^{k+1}\right| \end{array}\right]}_{\left(L+1\right)*N\times 1}$$

The iteration processes stop when the maximum value of the error vector $\Delta C_{max}^{k+1}$ is smaller than the tolerable error e (e.g., e = 10^−15^).

## Appendix D. Length of the Mass Transfer Zone ($X_{s}$) for the First Order Analytical Solution

Analytical solutions can be derived for the case of the first flooding of the column which are used here for verification of the numerical codes.

### Appendix D.1. Analytical Solution Based on the Film Diffusion Model

During the first flooding of the column, the front water flow is always contacting fresh contaminant material. Therefore, the solute concentration at the particle-water boundary is constant and in equilibrium with the solids:

(A25) $$C_{w,eq}=\frac{C_{s,ini}}{K_{d}}$$

Inserting Equation (A25) into Equation (7) gives:

(A26) $$\frac{\partial C_{w}}{\partial t}=k\frac{6\left(1-n\right)}{nd}\left(C_{w,eq}-C_{w}\right)$$

which upon integration yields the following analytical solution for the initial condition $C_{w\left(t=0\right)}=0$ (desorption):

(A27) $$\begin{array}{l} \overset{C_{w}}{\underset{0}{\int }}\frac{\partial C_{w}}{\left(C_{w,eq}-C_{w}\right)}=\overset{t}{\underset{0}{\int }}k\frac{6\left(1-n\right)}{nd}\partial t\\ -\ln\left(C_{w,eq}-C_{w}\right)+\ln\left(C_{w,eq}\right)=-\ln\left(1-\frac{C_{w}}{C_{w,eq}}\right)=k\frac{6\left(1-n\right)}{nd}t\\ \frac{C_{w}}{C_{w,eq}}=1-\exp\left(-k\frac{6\left(1-n\right)}{nd}t\right) \end{array}$$

The contact time in Equation (A27) can be substituted with the ratio of the travel distance (*x*) and the flow velocity (*v*). The length of the mass transfer zone is defined by setting the argument of the exponential function to −1, referring to the location x where the solute concentration in the groundwater reaches 63.2% of the equilibrium concentration.

(A28) $$X_{s,63.2\%}=\frac{v\;n\;d}{6k\left(1-n\right)}$$

Equation (A28) shows that the length of mass transfer zone depends on the flow velocity, inter-granular porosity as well as particle size, but is independent of the distribution coefficient.

If the length of the mass transfer zone is shorter than the column length ($X_{col}$), a concentration higher than 63.2% of the equilibrium concentration will be observed in the column effluent until the mass transfer zone arrives at the column outlet. The time needed to reach 63.2% equilibrium concentration at the column outlet equals:

(A29) $$\begin{array}{cc} t_{63.2\%} & =\frac{X_{s}}{v}+\frac{\left(X_{col}-X_{s}\right)}{v}R_{d}\\ & =\frac{X_{col}}{v}\left(1+K_{d}\frac{\rho_{b}}{n}\left(1-\frac{X_{s}}{X_{col}}\right)\right) \end{array}$$

Considering fast kinetics ($X_{s}\to 0$),$\;t_{63.2\%}$($\approx X_{col}/(v/R_{d})$) is mainly dominated by the retarded seepage velocity ($v/R_{d}$).

### Appendix D.2. Analytical Solution Based on the Intraparticle Pore Diffusion Model

Expressing internal mass transfer resistance by means of intraparticle pore diffusion, with mass transfer coefficient $k=D_{e}/\delta_{p},$ where $D_{e}$ is the effective intraparticle diffusion coefficient ($D_{e}=D_{aq}\varepsilon /\tau \approx D_{aq}$*ε^2^*) and the mean square displacement $\delta_{p}$ ($\delta_{p}=\sqrt{\pi D_{a}\;t_{c}})$ representing the diffusion distance, which grows with the square root of contact time between particles and water ($t_{c}$) at early times, leads to:

(A30) $$\frac{\partial C_{w}}{\partial t}=k\;A^{o}\left(C_{w,eq}-C_{w}\right)=\frac{D_{e}}{\sqrt{\pi D_{a}\;t_{c}}\;}\frac{6\left(1-n\right)}{nd}\left(C_{w,eq}-C_{w}\right)$$

The contact time between water and dry particles can be estimated by the ratio of particle size and flow velocity ($t_{c}=d/v$).

For the initial condition $C_{w\left(t=0\right)}=0$, integration of Equation (A30) yields the following analytical solution:

(A31) $$\begin{array}{l} \overset{C_{w}}{\underset{0}{\int }}\frac{\partial C_{w}}{\left(C_{w,eq}-C_{w}\right)}=\overset{t}{\underset{0}{\int }}\frac{D_{e}}{\sqrt{\pi D_{a}\;t_{c}}}\frac{6\left(1-n\right)}{nd}\partial t\\ -\ln\left(C_{w,eq}-C_{w}\right)+\ln\left(C_{w,eq}\right)=-\ln\left(1-\frac{C_{w}}{C_{w,eq}}\right)=\frac{D_{e}}{\sqrt{\pi D_{a}\frac{d}{v}\;}}\frac{6\left(1-n\right)}{nd}t\\ \frac{C_{w}}{C_{w,eq}}=1-\exp\left(-\frac{D_{e}}{\sqrt{\pi D_{a}\frac{d}{v}\;}}\frac{6\left(1-n\right)}{nd}t\right) \end{array}$$

The length of the mass transfer zone of intraparticle pore diffusion can be calculated by:

(A32) $$\begin{array}{cc} X_{s,63.2\%} & =\frac{v\;n\;d}{6\frac{D_{e}}{\sqrt{\pi D_{a}\frac{d}{v}\;}}\left(1-n\right)}\\ & =\sqrt{\frac{\pi d^{3}v}{D_{e}\left(\varepsilon +K_{d}\rho_{p}\right)}}\frac{n}{6\left(1-n\right)} \end{array}$$

$X_{s,63.2\%}$ based on intraparticle pore diffusion increases with particle size to the power of 3/2 ($d^{3/2}$) and decreases with the square root of the distribution coefficient ($\sqrt{K_{d}}$). The time required to observe the corresponding concentration of 63.2% of the equilibrium concentration at the column outlet can be determined with Equation (A29).

### Appendix D.3. Comparison of Analytical and Numerical Solution and Estimation of Mass Transfer Zone Length ($X_{s}$)

In Figure A2, analytical solutions for the increase of the concentration in the first water parcel over distance (which here represents time: t=x/v) during the first flooding of the column are shown for FD (Equation (A28)) and IPD (Equation (A31)). The numerical solutions are described in Appendix B and Appendix C.

A comparison reveals that the analytical solutions and the numerical solutions overlap almost perfectly for both FD and IPD (see Figure A2). This verifies the accuracy of the numerical model. The length of the mass transfer zone for FD is 0.35 cm and independent of $K_{d}$, and much shorter than for IPD with $X_{s}$ = 10 cm, 3.5 cm and 1.1 cm for $K_{d}$ values of 0.1 L kg^−1^, 1 L kg^−1^ and 10 L kg^−1^, respectively. The deviations between FD and IPD gradually vanish with increasing $K_{d}$ values. If the initial concentration in the column leachate is close to equilibrium, it may be used for the determination of $K_{d}$ ($K_{d}=C_{s,ini}/C_{w,peak}$); $K_{d}$ is overestimated if the initial effluent concentration does not reach equilibrium ($C_{w,peak}<C_{w,eq})$. The length of the mass transfer zone (Equations (A28) and (A32)) may be used to assess equilibrium at the beginning of the column test.

## Appendix E. Comparison of Analytical and Numerical Solution (Code Verification)

In order to further confirm the accuracy of the numerical solution, the initial concentration distribution in the column after first flooding, as well as leaching curves are compared with the analytical solution (Equation (6)). The analytical solution is only valid for equilibrium sorption conditions and to compare it with the numerical solution, fine particles ($d_{p,\;fine}$ = 63 μm) are used to get close to equilibrium (to fast FD kinetics). Figure A3 and Figure A4 show the good agreement of both solutions. The slight deviations between analytical and numerical solutions, especially at low $K_{d}$ values, are due to kinetics in the numerical solution. Deviations gradually vanish with the increase of $K_{d}$.

## References

[1] Röhler K., Haluska A.A., Susset B., Liu B., Grathwohl P. (2021). Long-term behavior of PFAS in contaminated agricultural soils in Germany. J. Contam. Hydrol..

[2] Löv Å., Larsbo M., Sjöstedt C., Cornelis G., Gustafsson J.P., Kleja D.B. (2019). Evaluating the ability of standardised leaching tests to predict metal(loid) leaching from intact soil columns using size-based elemental fractionation. Chemosphere.

[3] Inui T., Hori M., Takai A., Katsumi T., Zhan L., Chen Y., Bouazza A. (2019). Column percolation tests for evaluating the leaching behavior of marine sediment containing non-anthropogenic arsenic. Proceedings of the 8th International Congress on Environmental Geotechnics (from 28th October to 1st November 2018 in Hangzhou, China).

[4] Kalbe U., Bandow N., Bredow A., Mathies H., Piechotta C. (2014). Column leaching tests on soils containing less investigated organic pollutants. J. Geochem. Explor..

[5] Zhu Y., Hu Y., Guo Q., Zhao L., Li L., Wang Y., Hu G., Wibowo H., Francesco D.M. (2021). The effect of wet treatment on the distribution and leaching of heavy metals and salts of bottom ash from municipal solid waste incineration. Environ. Eng. Sci..

[6] Kumar A., Samadder S.R., Kumar V. (2019). Assessment of groundwater contamination risk due to fly ash leaching using column study. Environ. Earth Sci..

[7] Chan W.P., Ren F., Dou X., Yin K., Chang V.W.C. (2018). A large-scale field trial experiment to derive effective release of heavy metals from incineration bottom ashes during construction in land reclamation. Sci. Total Environ..

[8] Di Gianfilippo M., Costa G., Verginelli I., Gavasci R., Lombardi F. (2016). Analysis and interpretation of the leaching behaviour of waste thermal treatment bottom ash by batch and column tests. Waste Manag..

[9] Tsiridis V., Petala M., Samaras P., Sakellaropoulos G.P. (2015). Evaluation of interactions between soil and coal fly ash leachates using column percolation tests. Waste Manag..

[10] Lange C.N., Flues M., Hiromoto G., Boscov M.E.G., Camargo I.M.C. (2019). Long-term leaching of As, Cd, Mo, Pb, and Zn from coal fly ash in column test. Environ. Monit. Assess..

[11] Liu B., Li J., Wang Z., Zeng Y., Ren Q. (2020). Long-term leaching characterization and geochemical modeling of chromium released from AOD slag. Environ. Sci. Pollut. Res..

[12] Schwab O., Bayer P., Juraske R., Verones F., Hellweg S. (2014). Beyond the material grave: Life Cycle Impact Assessment of leaching from secondary materials in road and earth constructions. Waste Manag..

[13] Diotti A., Galvin A.P., Piccinali A., Plizzari G., Sorlini S. (2020). Chemical and leaching behavior of construction and demolition wastes and recycled aggregates. Sustainability.

[14] Liu H., Zhang J., Li B., Zhou N., Xiao X., Li M., Zhu C. (2020). Environmental behavior of construction and demolition waste as recycled aggregates for backfilling in mines: Leaching toxicity and surface subsidence studies. J. Hazard. Mater..

[15] Bandow N., Finkel M., Grathwohl P., Kalbe U. (2019). Influence of flow rate and particle size on local equilibrium in column percolation tests using crushed masonry. J. Mater. Cycles Waste Manag..

[16] Butera S., Hyks J., Christensen T.H., Astrup T.F. (2015). Construction and demolition waste: Comparison of standard up-flow column and down-flow lysimeter leaching tests. Waste Manag..

[17] Chen Z., Zhang P., Brown K., Branch J., van der Sloot H., Meeussen J., Delapp R., Um W., Kosson D. (2021). Development of a geochemical speciation model for use in evaluating leaching from a cementitious radioactive waste form. Environ. Sci. Technol..

[18] Molleda A., López A., Cuartas M., Lobo A. (2020). Release of pollutants in MBT landfills: Laboratory versus field. Chemosphere.

[19] Grathwohl P., van der Sloot H., Quevauviller P. (2007). Groundwater risk assessment at contaminated sites (GRACOS): Test methods and modelling approaches. Groundwater Science and Policy.

[20] Susset B., Grathwohl P. (2011). Leaching standards for mineral recycling materials—A harmonized regulatory concept for the upcoming German Recycling Decree. Waste Manag..

[21] Grathwohl P., Susset B. (2009). Comparison of percolation to batch and sequential leaching tests: Theory and data. Waste Manag..

[22] Grathwohl P. (2014). On equilibration of pore water in column leaching tests. Waste Manag..

[23] Grathwohl P., Reinhard M. (1993). Desorption of Trichloroethylene in Aquifer Material: Rate Limitation at the Grain Scale. Environ. Sci. Technol..

[24] Rügner H., Kleineidam S., Grathwohl P. (1999). Long term sorption kinetics of phenanthrene in aquifer materials. Environ. Sci. Technol..

[25] Wang G., Kleineidam S., Grathwohl P. (2007). Sorption/desorption reversibility of phenanthrene in soils and carbonaceous materials. Environ. Sci. Technol..

[26] Finkel M., Grathwohl P. (2017). Impact of pre-equilibration and diffusion limited release kinetics on effluent concentration in column leaching tests: Insights from numerical simulations. Waste Manag..

[27] Kleineidam S., Rügner H., Grathwohl P. (1999). Impact of grain scale heterogeneity on slow sorption kinetics. Environ. Toxicol. Chem..

[28] Jäger R., Liedl R. (2000). Prognose der Sorptionskinetik organischer Schadstoffe in heterogenem Aquifermaterial (Predicting sorption kinetics of organic contaminants in heterogeneous aquifer material). Grundwasser.

[29] Xiao Y., Feng Z., Huang X., Huang L., Chen Y., Wang L., Long Z. (2015). Recovery of rare earths from weathered crust elution-deposited rare earth ore without ammonia-nitrogen pollution: I. leaching with magnesium sulfate. Hydrometallurgy.

[30] Yin K., Chan W.P., Dou X., Lisak G., Chang V.W.C. (2020). Kinetics and modeling of trace metal leaching from bottom ashes dominated by diffusion or advection. Sci. Total Environ..

[31] Ogata A., Banks R.B. (1961). A solution of the differential equation of longitudinal dispersion in porous media. Professional Paper.

[32] Boving T., Grathwohl P. (2001). Matrix diffusion coefficients in sandstones and limestones: Relationship to permeability and porosity. J. Contam. Hydrol..

[33] Kalbe U., Berger W., Eckardt J., Simon F.G. (2008). Evaluation of leaching and extraction procedures for soil and waste. Waste Manag..

[34] Kalbe U., Berger W., Simon F.G., Eckardt J., Christoph G. (2007). Results of interlaboratory comparisons of column percolation tests. J. Hazard. Mater..

[35] Liu Y., Illangasekare T.H., Kitanidis P.K. (2014). Long-term mass transfer and mixing-controlled reactions of a DNAPL plume from persistent residuals. J. Contam. Hydrol..

[36] Naka A., Yasutaka T., Sakanakura H., Kalbe U., Watanabe Y., Inoba S., Takeo M., Inui T., Katsumi T., Fujikawa T. (2016). Column percolation test for contaminated soils- Key factors for standardization. J. Hazard. Mater..

[37] Arters D.C., Fan L.S. (1990). Experimental methods and correlation of solid-liquid mass transfer in fluidized beds. Chem. Eng. Sci..

[38] Blasius H. (1908). Grenzschichten in Flüssigkeiten mit kleiner Reibung (The boundary layers in fluids with little friction). J. Appl. Math. Phys..

[39] Calderbank P.H., Moo-Young M.B. (1995). The continuous phase heat and mass transfer properties of dispersions. Chem. Eng. Sci..

[40] Kirwan J., Armenante P.M. (1989). Mass transfer to microparticle systems. Chem. Eng. Sci..

[41] Levins D.M., Glastonbury J.R. (1972). Application of Kolmogorofff’s theory to particle-liquid mass transfer in agitated vessels. Chem. Eng. Sci..

[42] Mao H., Chisti Y., Moo-Young M. (1992). Multiphase hydrodynamics and solid-liquid mass transfer in an external-loop airlift reactor- a comparative study. Chem. Eng. Commun..

[43] Ohashi H., Sugawara T., Kikuchi K.I. (1981). Mass transfer between particles and liquid in solid-liquid two-phase flow through vertical tubes. J. Chem. Eng. Jpn..

[44] Sherwood T.K., Pigford R.L., Wilke R.L. (1975). “Mass Transfer”.

[45] Cirpka O.A. (2020). Solute and heat transport. Lecture Notes “Environmental Modeling 2”.

[46] Liedl R., Ptak T. (2003). Modelling of diffusion-limited retardation of contaminants in hydraulically and lithologically nonuniform media. J. Contam. Hydrol..

