# Supplementary material for: Mass Transfer Principles in Column Percolation Tests: Initial Conditions and Tailing in Heterogeneous Materials
Source: Materials (Basel). 2021 Aug 20;14(16):4708. doi: 10.3390/ma14164708 (PMC8398455; doi:10.3390/ma14164708)
Supplement: Supplementary file 1 [file materials-14-04708-s001.zip › materials-1324807-supplementary proof done.pdf]

materials

MDPI

Supplementary Material

Mass Transfer Principles in Column Percolation Tests: Initial Conditions and Tailing in Heterogeneous Materials

Binlong Liu, Michael Finkel and Peter Grathwohl *

Center for Applied Geoscience, University of Tübingen, Schnarrenbergstraße 94-96, 72076 Tübingen, Germany; binlong.liu@uni-tuebingen.de (B.L.); michael.finkel@uni-tuebingen.de (M.F.)

* Correspondence: grathwohl@uni-tuebingen.de

The following figures (S1 – S8) compare leaching scenarios without and with dispersion.

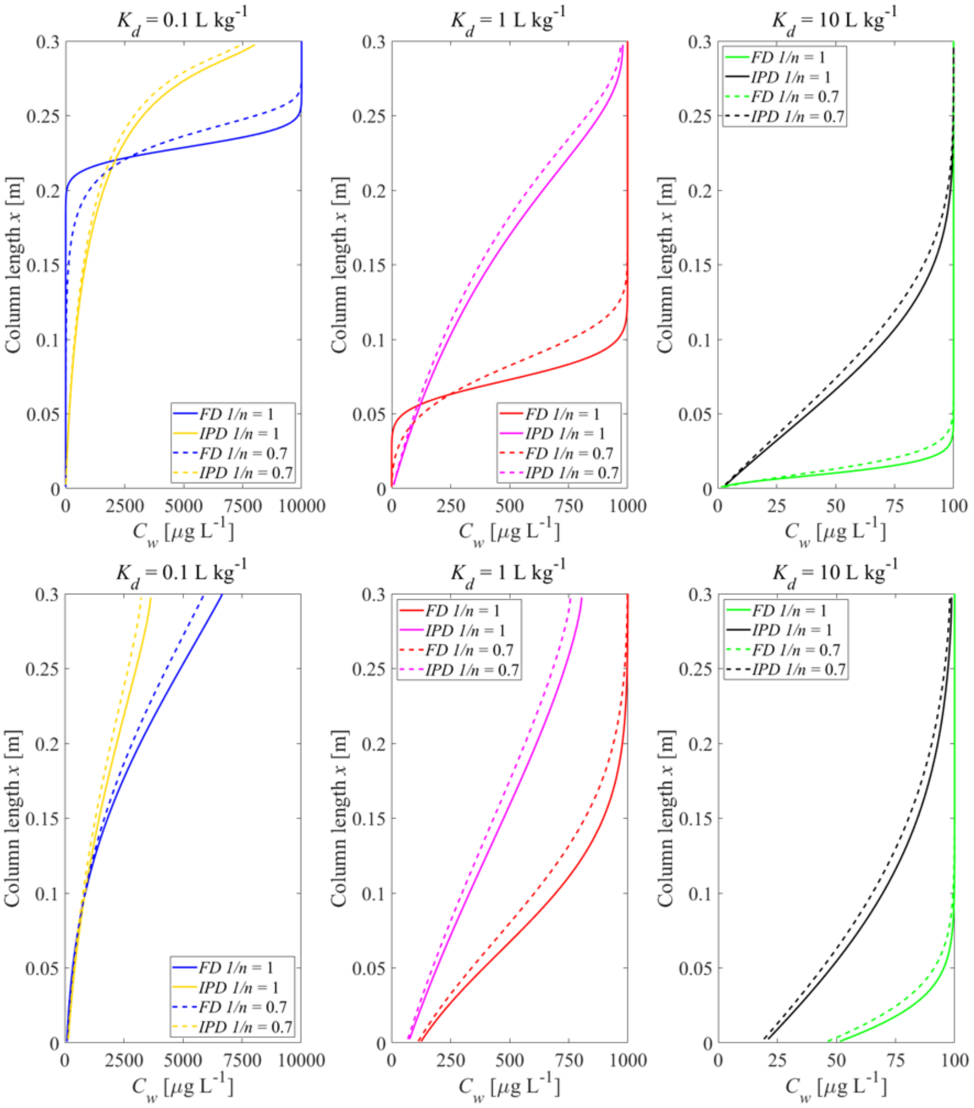

Figure S1. Initial concentration profiles in the column after the first flooding (up-flow) without and with dispersion (top and bottom panel); solid lines: linear sorption; dashed lines: non-linear sorption cases (based on a Freundlich exponent $1/n$ = 0.7), n = 0.45, v = 1.67 × 10^{-5} m s^{-1}, α/x = 0 or 0.1, $C_{s,ini}$ = 1000 μg kg^{-1}, t_c = 5 h, D_{aq} = 1 × 10^{-9} m^2 s^{-1}, ε = 0.05, $d_{p,coarse}$ = 2000 μm.

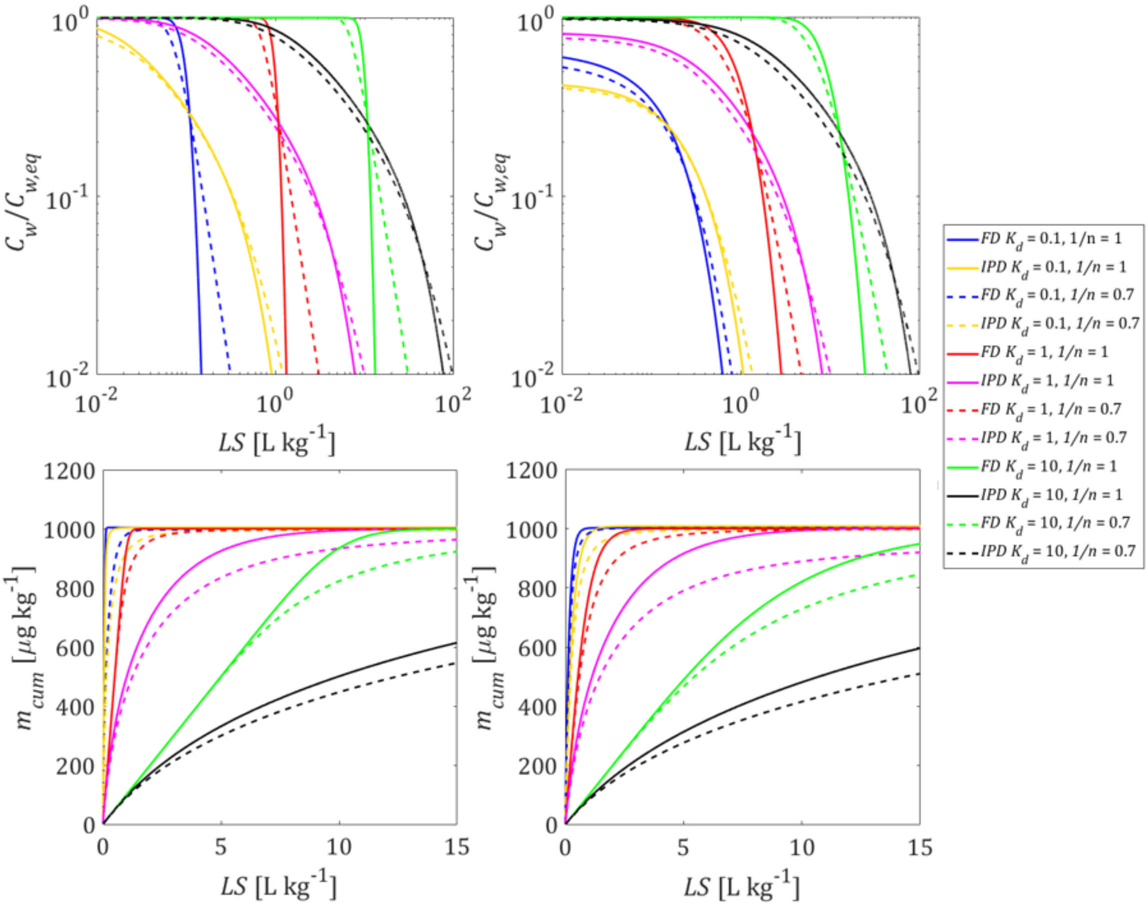

Figure S2. Normalized concentrations ($C_w/C_{w,eq}$) as well as cumulative concentrations (m_{cum}) in the column effluent vs. time (expressed as liquid to solid ratio: LS) for different initial conditions depicted in Figure S1; solid lines: linear sorption; dashed lines: nonlinear sorption. Left column: without dispersion; right column: with dispersion.

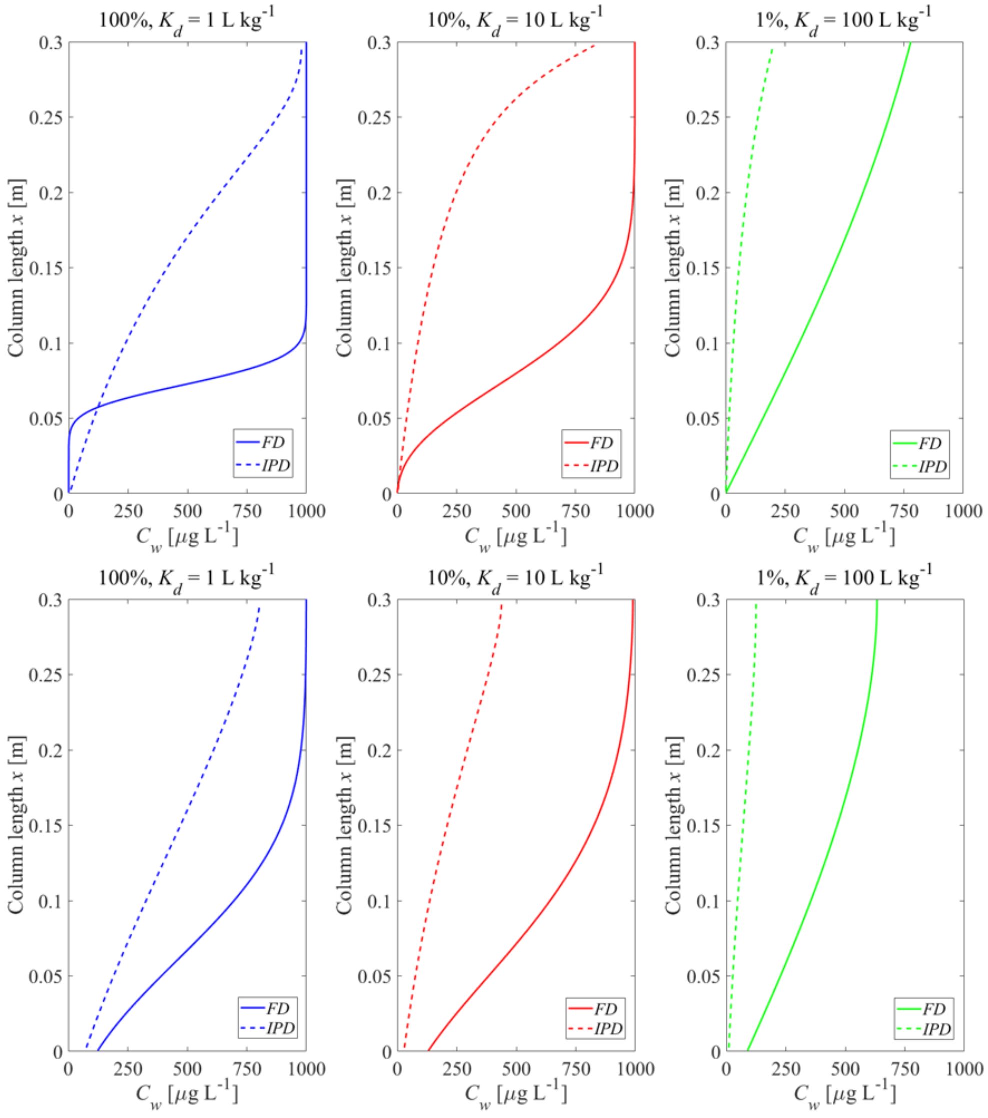

Figure S3. Initial concentration distribution in the column after the first flooding (up-flow) for different bi-modal compositions of sorbing and non-sorbing particles; left column: homogeneous case with average K_d (= $K_{d,av}$ = 1 L kg^{-1}); mid column: only 10% of the particles carry the contaminant at K_d = 10 × $K_{d,av}$; right column: only 1% of the particles carry the contaminant at K_d = 100 × $K_{d,av}$; the average $K_{d,av}$ of the entire material is the same for all compositions; solid lines: film diffusion case, dashed lines: intraparticle diffusion case. Top panel: without dispersion; bottom panel: with dispersion; n = 0.45, v = 1.67 × 10^{-5} m s^{-1}, α/x = 0 or 0.1, $C_{s,ini}$ = 1000 µg kg^{-1}, t_c = 5 h, D_{aq} = 1 × 10^{-9} m^2 s^{-1}, ε = 0.05, $d_{p,coarse}$ = 2000 µm.

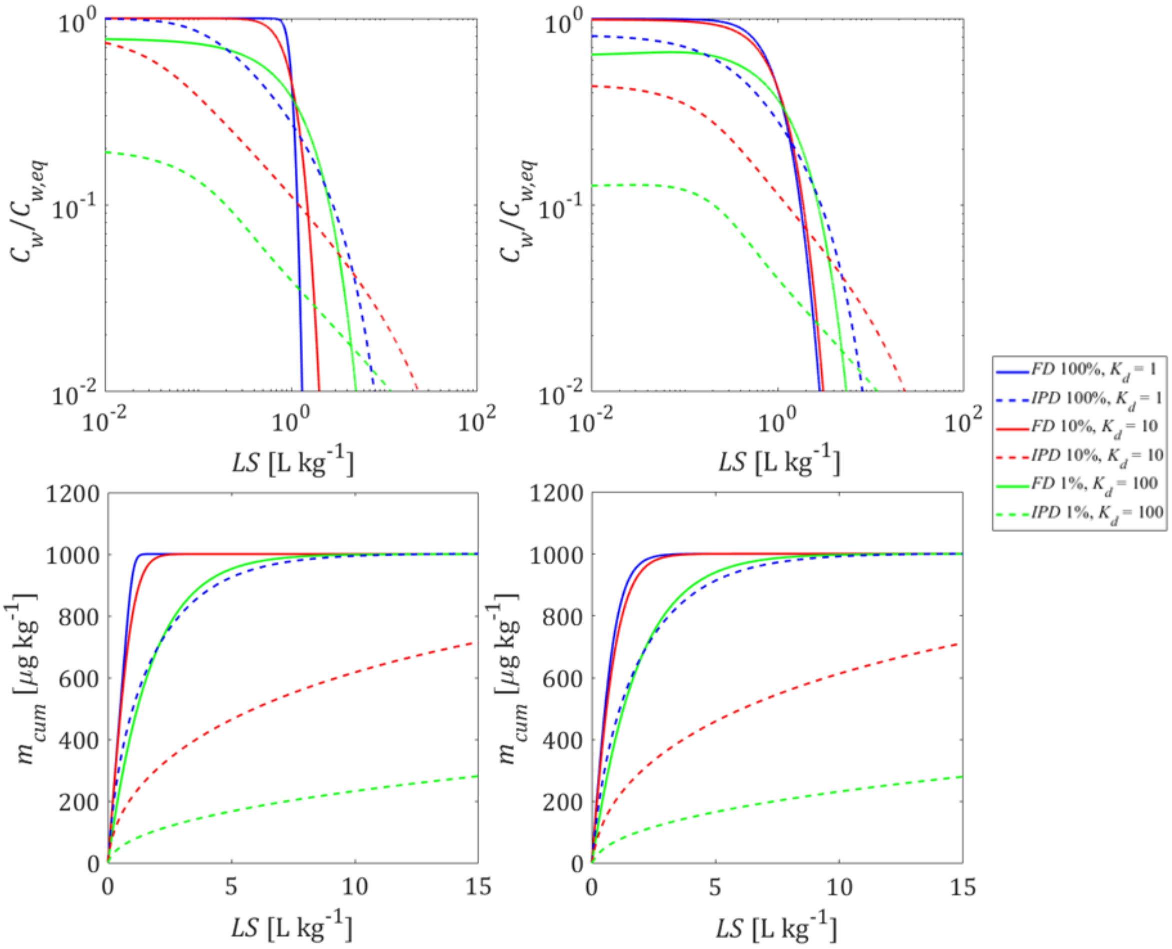

Figure S4. Normalized concentrations ($C_w/C_{w,eq}$) as well as cumulative concentrations (m_{cum}) in the column effluent vs. time (expressed as liquid to solid ratio: *LS*) for different combinations of sorbing particles and distribution coefficients (initial conditions depicted in Figure S3); left: without dispersion; right: with dispersion; solid lines: film diffusion cases, dashed lines: intraparticle diffusion cases.

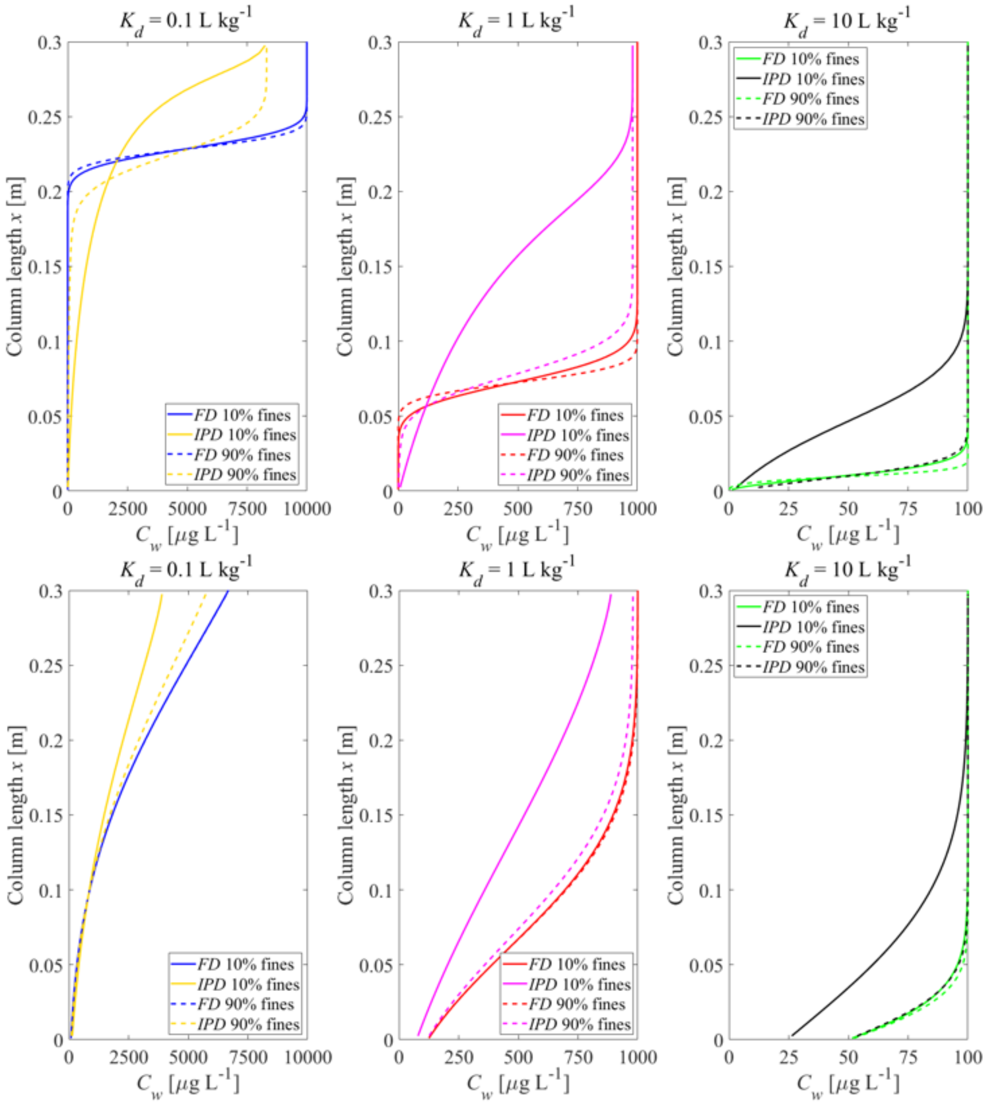

Figure S5. Initial concentration distribution in the column after the first flooding (up-flow) for two different bi-modal grain size distributions of fine and coarse particles; solid lines: fine particle mass fraction 10%; dashed lines: fine particle mass fraction 90%. (n = 0.45, v = 1.67 × 10^{-5} m s^{-1}, α/x = 0 or 0.1, $C_{s,ini}$ = 1000 µg kg^{-1}, t_c = 5 h, D_{aq} = 1 × 10^{-9} m^2 s^{-1}, ε = 0.05, $d_{p,coarse}$ = 2000 µm, $d_{p,fine}$ = 63 µm); top panel: without dispersion; bottom panel: with dispersion.

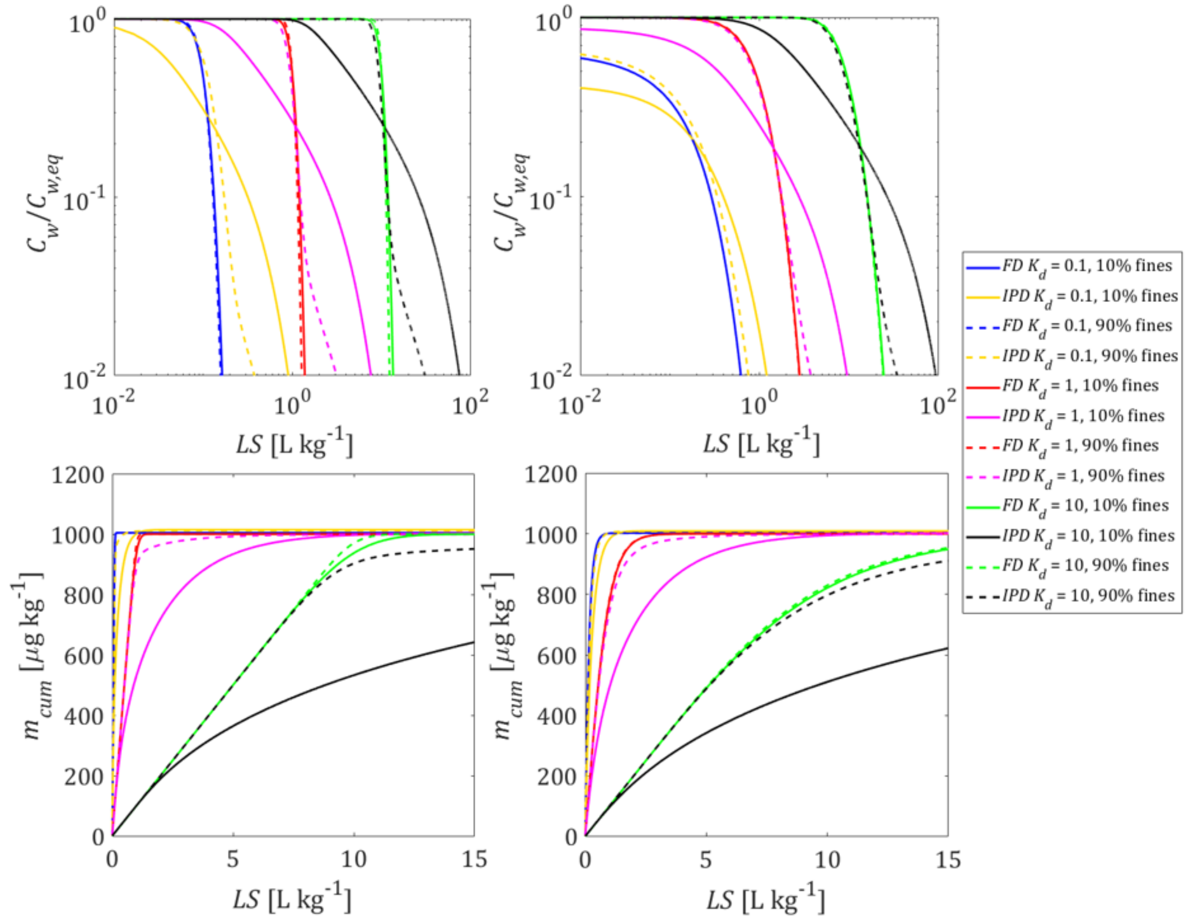

Figure S6. Influence of different grain size fractions and distribution coefficients on normalized concentrations ($C_w/C_{w,eq}$) as well as cumulative concentrations (m_{cum}) in the column effluent vs. time (expressed as liquid to solid ratio *LS*); left: without dispersion; right: with dispersion; solid lines: fine particle mass fraction 10%; dashed lines: fine particle mass fraction 90%; kinetic parameters are the same as Figure S5.

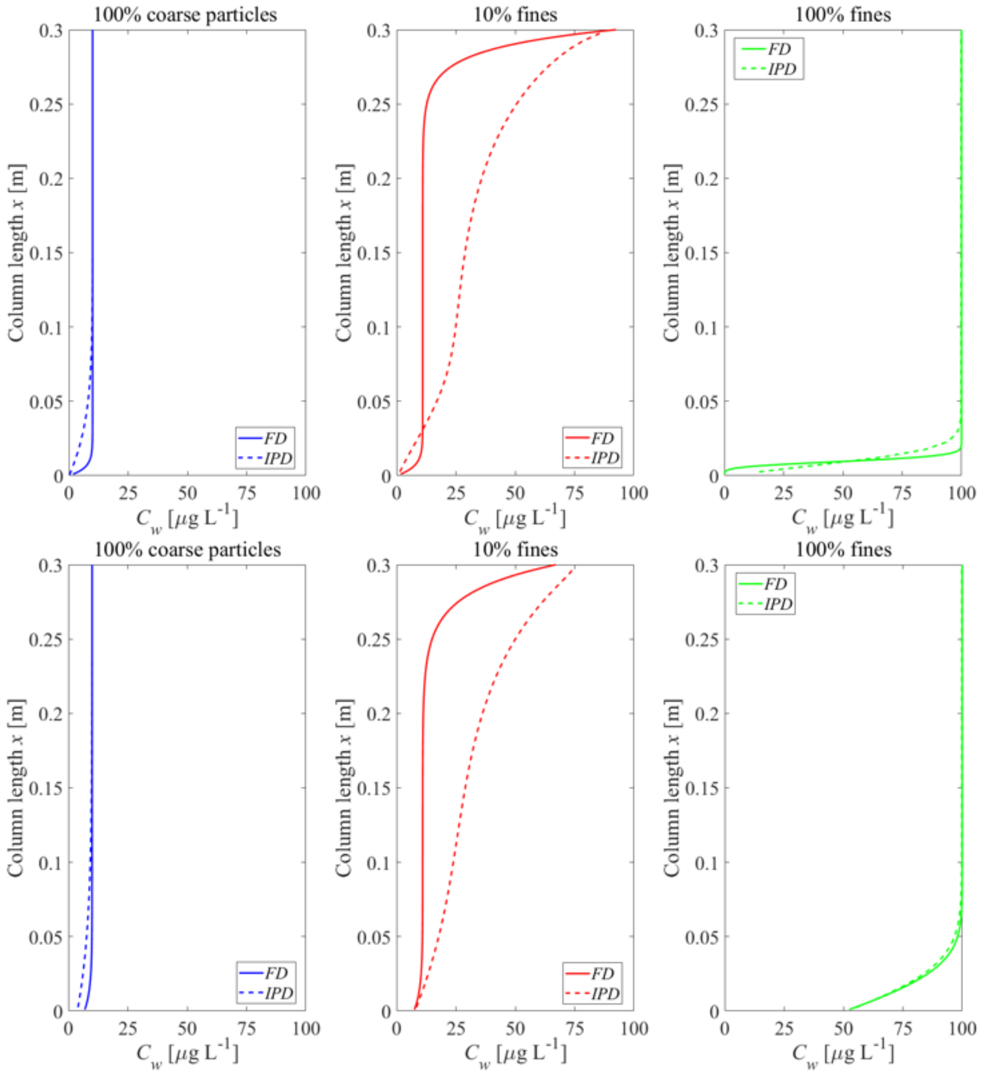

Figure S7. Initial concentration distribution in the column after the first flooding (up-flow) for different bi-modal material compositions of fine particles with low sorption capacity (K_d = 10 L kg^{-1}) and coarse particles with high sorption capacity; left: 100% coarse particles (K_d = 100 L kg^{-1}); middle: mixed sample with 10% fine particles; right: 100% fine particles; solid lines: film diffusion (FD), dashed lines: intraparticle diffusion cases (IPD); n = 0.45, v = 1.67 × 10^{-5} m s^{-1}, α/x = 0 or 0.1, $C_{s,ini}$ = 1000 μg kg^{-1}, t_c = 5 h, D_{aq} = 1 × 10^{-9} m^2 s^{-1}, ε = 0.05, $d_{p,coarse}$ = 2000 μm, $d_{p,fine}$ = 63 μm; top panel: without dispersion; bottom panel: with dispersion.

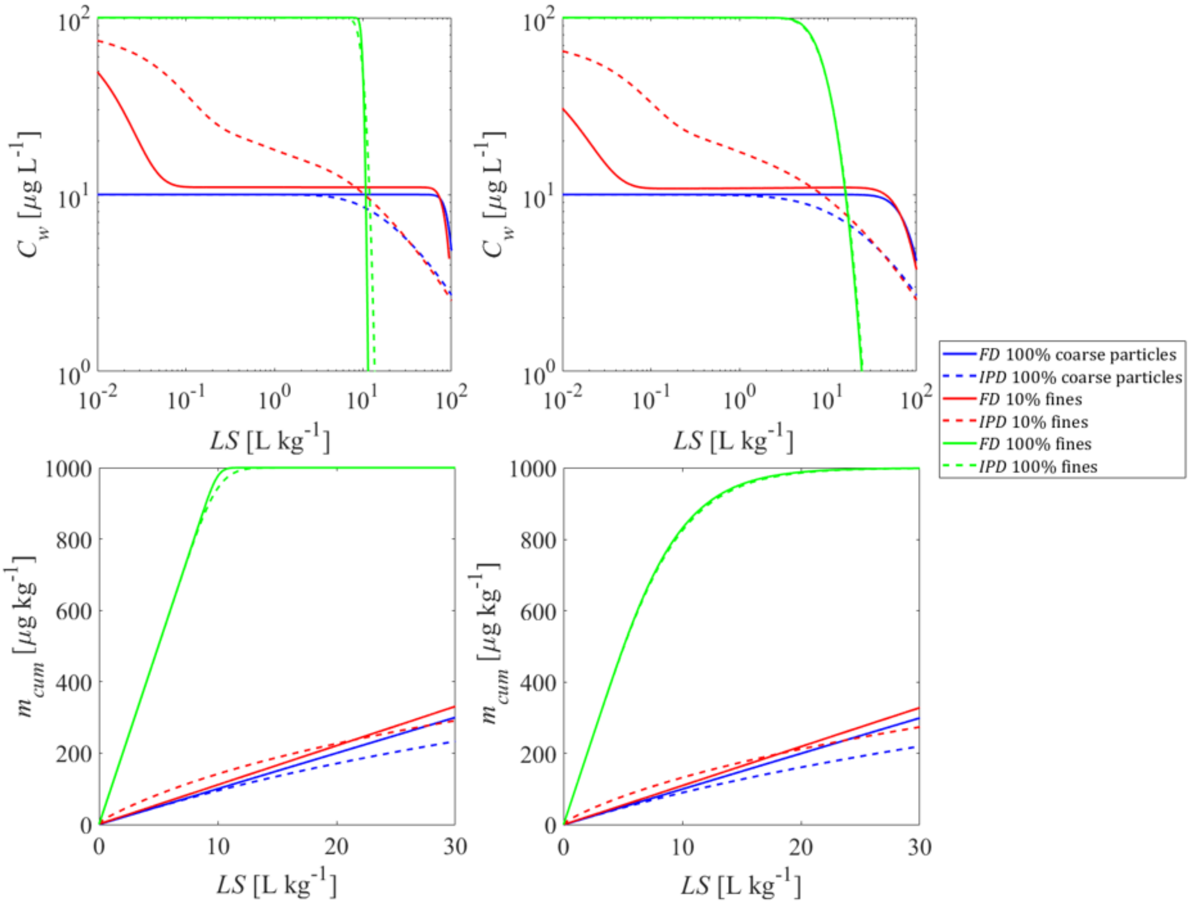

Figure S8. Leachate concentrations (C_w) as well as cumulative concentrations (m_{cum}) in the column effluent vs. time (expressed as liquid to solid ratio: *LS*) for different combinations of fine particles with low sorption capacity (K_d = 10 L kg^{-1}) and coarse particles with high sorption capacity (K_d = 100 L kg^{-1}); left: without dispersion; right: with dispersion; solid lines: film diffusion cases, dashed lines: intraparticle diffusion cases; kinetic parameters are the same as Figure S7.
